# Chromosome-wide mapping of DNA methylation patterns in normal and malignant prostate cells reveals pervasive methylation of gene-associated and conserved intergenic sequences

**DOI:** 10.1186/1471-2164-12-313

**Published:** 2011-06-13

**Authors:** Srinivasan Yegnasubramanian, Zhijin Wu, Michael C Haffner, David Esopi, Martin J Aryee, Raghav Badrinath, Tony L He, James D Morgan, Benilton Carvalho, Qizhi Zheng, Angelo M De Marzo, Rafael A Irizarry, William G Nelson

**Affiliations:** 1 Sidney Kimmel Comprehensive Cancer Center, Johns Hopkins University School of Medicine, Baltimore, Maryland, USA; 2 Department of Pathology, Johns Hopkins University School of Medicine, Baltimore, Maryland, USA; 3 Department of Biostatistics, Johns Hopkins University Bloomberg School of Public Health, Baltimore, Maryland, USA; 4 Center for Statistical Science, Brown University, Providence, Rhode Island, USA

**Keywords:** DNA methylation, prostate cancer, tiling microarray, epigenetics, methylated DNA binding domain, MBD-chip, ADAMTS1, SCARF2, DSCR9, HLCS

## Abstract

**Background:**

DNA methylation has been linked to genome regulation and dysregulation in health and disease respectively, and methods for characterizing genomic DNA methylation patterns are rapidly emerging. We have developed/refined methods for enrichment of methylated genomic fragments using the methyl-binding domain of the human MBD2 protein (MBD2-MBD) followed by analysis with high-density tiling microarrays. This MBD-chip approach was used to characterize DNA methylation patterns across all non-repetitive sequences of human chromosomes 21 and 22 at high-resolution in normal and malignant prostate cells.

**Results:**

Examining this data using computational methods that were designed specifically for DNA methylation tiling array data revealed widespread methylation of both gene promoter and non-promoter regions in cancer and normal cells. In addition to identifying several novel cancer hypermethylated 5' gene upstream regions that mediated epigenetic gene silencing, we also found several hypermethylated 3' gene downstream, intragenic and intergenic regions. The hypermethylated intragenic regions were highly enriched for overlap with intron-exon boundaries, suggesting a possible role in regulation of alternative transcriptional start sites, exon usage and/or splicing. The hypermethylated intergenic regions showed significant enrichment for conservation across vertebrate species. A sampling of these newly identified promoter (*ADAMTS1 *and *SCARF2 *genes) and non-promoter (downstream or within *DSCR9*, *C21orf57 *and *HLCS *genes) hypermethylated regions were effective in distinguishing malignant from normal prostate tissues and/or cell lines.

**Conclusions:**

Comparison of chromosome-wide DNA methylation patterns in normal and malignant prostate cells revealed significant methylation of gene-proximal and conserved intergenic sequences. Such analyses can be easily extended for genome-wide methylation analysis in health and disease.

## Background

Methylation at the 5-position of cytosine in CpG dinucleotides is a key epigenetic process in vertebrate species where it serves critical roles in normal genome homeostasis, including transcriptional regulation, establishment of chromatin structure, suppression of repetitive elements, imprinting, and × chromosome inactivation [1,2]. Furthermore, DNA methylation defects are a hallmark of many human diseases including cancer [3]. Characterizing DNA methylation patterns genome-wide and with high-resolution can yield many insights into human health and disease and could provide novel DNA-based biomarkers for detection and risk stratification of various human health disorders. Such DNA based biomarkers are already entering clinical use for detection of various cancers including prostate cancer [4].

Current methods for genome-wide DNA methylation analysis differentiate between methylated and unmethylated DNA on the basis of sodium bisulfite modification, methylation-sensitive (e.g. R.HpaII) and -specific (e.g. R.McrBcI) restriction enzymes, and/or affinity reagents specific for methylated DNA such as the anti-5meC antibody or recombinant methyl-binding domain (MBD) polypeptides [5,6]. Among these, the affinity-based strategies are particularly attractive because they are cost-effective, are not limited to specific target sequences, generate a positive signal for methylated DNA, and can be highly effective in fractionating methylated DNA from unmethylated DNA. In particular, the MBD approaches, pioneered by Adrian Bird and colleagues [7,8], are highly effective because the MBD polypeptides can recognize 5meC in double stranded DNA unlike the currently available antibodies. Even among the MBD polypeptides, just the MBD domain of the human MBD2 protein (MBD2-MBD) has exquisitely high affinity and specificity for 5meC, and previous reports have used this reagent to sensitively and specifically detect methylated DNA from as few as 5 cell equivalents [9].

**Figure 1:**
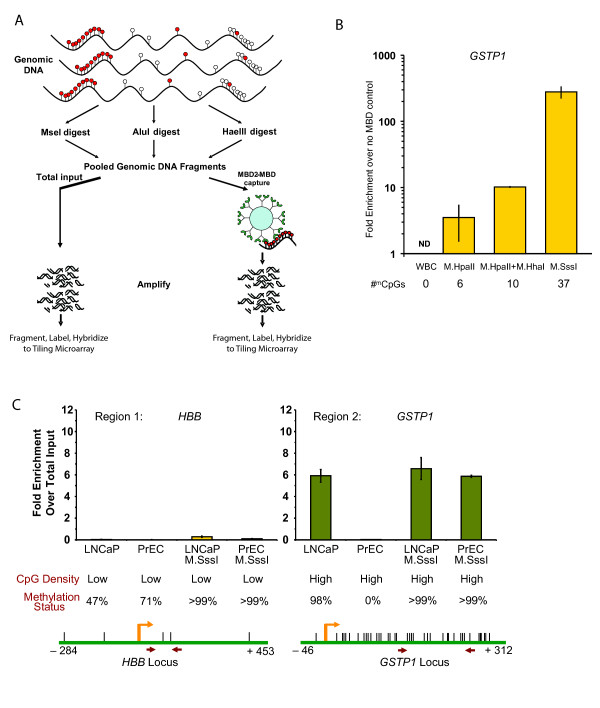
**Overview and pre-microarray performance of MBD-chip**. **A**, Overview of MBD-chip. Genomic DNA is: i) fragmented (in this case with restriction enzymes), ii) enriched for methylated DNA using MBD2-MBD-magnetic beads, and iii) amplified, fragmented, labeled and hybridized to tiling microarrays. Comparison with a total input fraction allows identification of methylated regions. **B**, Degree of MBD2-MBD enrichment is non-linearly proportional to the number of methylated CpGs. WBC DNA was methylated at 0, 6, 10 or 37 CpG sites within an R.AluI restriction fragment within the *GSTP1 *promoter by treatment with M.HpaII +/- M.HhaI or with M.SssI or no enzymes. The degree of MBD2-MBD enrichment compared to mock (no MBD control), as measured by qPCR, was related to the number of methylated CpGs. ND, not detectable. **C**, The MBD-chip process enriches DNA with high density of methylated-CpGs. DNA from LNCaP and PrEC cells was completely methylated with M.SssI or left untreated. MBD2-MBD enrichment at regions in *HBB *and *GSTP1 *promoters, as examined by qPCR, are shown. The CpG density (Low, indicates < 5 CpGs per kbp and high indicates > 20 CpGs per kbp) and known degree of methylation (as determined by the Infinium 27K DNA methylation platform for *HBB *(unpublished data; Yegnasubramanian S and Haffner MC, 2011) and by bisulfite sequencing for *GSTP1 *[9]) are indicated. Schematics of each region are annotated with position of CpGs (vertical hashes), transcriptional start sites (yellow arrow), and amplification primers (red arrows). The start and end positions are with respect to transcriptional start sites.

An emerging strategy for analysis of DNA fractionated by affinity-based enrichment has been to hybridize enriched libraries to promoter [10-12], CpG island [13-15], chromosome-wide [15], or genome-wide [16] tiling microarrays or to analyze by next generation sequencing [17,18]. Here, we describe the use of an MBD-chip approach (Figure 1A) to compare the chromosome-wide DNA methylation patterns in LNCaP prostate cancer cells and PrEC normal prostate epithelial cells. Using this information, we make novel observations regarding cancer-normal differences in methylation patterns in various biologically meaningful genome compartments without bias to promoter regions. This method uses MBD2-MBD bound magnetic beads to specifically enrich for methylated DNA fragments followed by processing, hybridization and analysis with high-density, oligonucleotide tiling microarrays containing probes interrogating all non-repetitive sequences on chromosomes 21 and 22 with an average interval between probes of 10 base pairs (bp). We also present novel analytical strategies to overcome challenges in pre-processing and analysis of DNA methylation microarray data and approaches for biological interpretation of such data. These analyses revealed pervasive methylation of both gene promoter and non-promoter regions in cancer and normal cells. Focusing on the differentially methylated regions between cancer and normal cells, hypermethylated non-promoter regions include intragenic and intergenic regions. The hypermethylated intragenic regions were highly enriched for localization to exons and intron-exon boundaries, suggesting a possible role in regulation of alternative transcriptional start sites, exon usage and/or splicing. The hypermethylated intergenic regions showed a high degree of enrichment for conservation across vertebrate species. Regardless of their regulatory role, these intra- and intergenic hypermethylated regions, in addition to the promoter hypermethylated regions, could be used to distinguish prostate cancer from normal prostate and therefore could serve as biomarkers for prostate cancer detection.

## Results

### Development and refinement of MBD-Chip and associated computational analyses

We previously showed that MBD2-MBD polypeptide-bound magnetic beads could be used to efficiently and quantitatively capture methylated DNA fragments [9]. To further characterize the binding properties of the MBD2-MBD magnetic beads, we used human white blood cell (WBC) genomic DNA known not to be methylated at the *GSTP1 *promoter, and treated it with the M.HhaI (5'-GCGC-3' recognition sites) and/or M.HpaII (5'-CCGG-3' recognition sites) methyltransferases or with M.SssI (5'-CG-3' recognition sites) methyltransferase or mock (no enzyme) to produce genomic DNA that contains 0, 6, 10, or 37 methylated CpGs within a 262 bp *GSTP1 *R.AluI fragment. Subjecting these R.AluI digested DNAs to MBD2-MBD enrichment and quantifying the amount of enriched *GSTP1 *promoter DNA by real time PCR revealed that the degree of enrichment was proportional to the number of methylated CpGs in a nonlinear fashion (Figure 1B). Next, we assessed the performance of the MBD2-MBD enrichment at two gene promoters (*HBB, GSTP1*) with known methylation patterns in LNCaP and PrEC cells [9,19]. The genomic DNAs were either completely methylated with the M.SssI methyltransferase or left untreated. These analyses further confirmed that the MBD2-MBD enrichment robustly captures densely methylated regions, but not regions lacking methylated CpG dinucleotides or regions with very low density of CpG dinucleotides (Figure 1C).

To analyze chromosome-wide patterns of methylation, we used an MBD-chip approach in which genomic DNA from LNCaP and PrEC cells was first fragmented and divided into a total input fraction and an enriched methylated fraction. The enriched methylated fraction was subjected to enrichment for methylated fragments using MBD2-MBD bound magnetic beads while the total input fraction was not subjected to enrichment. Each fraction was then amplified by random-primed PCR (R-PCR), fragmented further, end-labeled, and hybridized to microarrays containing probes interrogating all non-repetitive sequences on human chromosomes 21 and 22 with an average inter-probe separation of 10 bp [20]. Each sample was analyzed in duplicate experiments.

We next explored pre-processing of the DNA methylation microarray data to facilitate downstream analysis of absolute and differential methylation in the LNCaP and PrEC specimens. As with nearly all oligonucleotide microarray platforms, the Affymetrix tiling arrays exhibited strong probe-effects, in which different probes on the tiling array have inherent differences in their behavior even when the underlying biological signal is known to be constant. Because the total input fraction should theoretically have uniform biological signal across all probes on the tiling arrays, we took a log-ratio of the enriched fraction to the total input to correct for these probe effects.

It is well known from chromatin immunoprecipitation microarray (ChIP-chip) experiments that even after taking a log-ratio to the total input fraction, significant residual sequence-based effects can persist. In one popular analytical approach called model-based analysis of tiling arrays (MAT), it is assumed that the majority of genomic regions should not be enriched, and data across all probes is used to build a sequence-based model to account for these residual sequence-based effects [21]. Unfortunately, a direct application of this method to the methylation tiling array data may be suboptimal since it may not be valid to assume that the majority of the genome is not methylated. However, it is known that in adult somatic cells, DNA methylation is almost entirely restricted to CpG dinucleotides [22]. This, combined with our empirical observations that the MBD2-MBD enrichment does not retain DNA fragments with very low CpG density, allows us to make the assumption that regions of the genome with very low CpG density should not be enriched and that any signal arising from such regions in the genome are due to spurious effects. Therefore, to assess residual sequence-based effects, we defined "null probes" as those interrogating regions of chromosomes 21 and 22 with extremely low CpG density (< 4 CpG's per 1000 bp). No appreciable enrichment signal is expected on these probes due to lack of CpGs. Interestingly, we noted a strong residual probe-enrichment interaction effect in which the log-ratio steadily increased with probe guanine and cytosine (GC) content (Additional File 1 panel A). Additionally, likely because the GC content in probes interrogating proximal genomic segments is expected to be similar, we also observed a strong positional autocorrelation in the data from these regions (Additional File 1 panel B). We adjusted for this GC content bias by subtracting a baseline log-ratio for each GC stratum estimated from the null probes as defined above. This adjustment resulted in baseline normalization of the samples with the log-ratio in unmethylated regions set to zero and, interestingly, essentially eliminated the autocorrelation in null probes (Additional File 1 panel B). To improve stability, we next smoothed these adjusted log-ratios by taking a running median across k = 7 consecutive probes (since the tiling interval is 35 bp on average, the smoothing window is ~250 bp, approximately equivalent to the modal fragmentation size), where k is a smoothing parameter. This smoothed adjusted log-ratio demonstrated a distribution that highly resembled what would be expected for an independent and identically distributed normal distribution (Additional File 1 panel C), thus facilitating straight-forward statistical inference. Therefore, this simple within-sample procedure allowed baseline normalization across samples, elimination of spurious GC content based effects, elimination of positional autocorrelation, and the ability to assign statistical significance to a given region. Additional File 2 panel A shows the effects of our pre-processing approach for a representative 15 kilobase pair (kbp) region of chromosome (Chr) 21 in LNCaP cells. Note that the final adjusted smoothed log-ratios have attenuated many questionable signals seen in the raw and smoothed log-ratios while preserving the signal at specific regions. The accuracy of the final adjusted smoothed log-ratios was confirmed by bisulfite sequencing of representative regions (see Additional Files 2, 3 and 4 and figures referenced in different sections below).

Using an empirical normal distribution defined using the null probes, we could then calculate a Z-score for each probe on the array. The Z-score represents the number of standard deviations separating the smoothed adjusted log-ratio of a probe from the median of the null probes having the same GC content. Methylated regions were defined as those regions in which the additive Z-score across all probes within 250 bp windows was >4, and at least one probe had Z > 3. We then merged all windows separated by < 250 bp together and calculated the additive Z-score of these merged regions and ranked them by this Z-score in order to highlight larger regions when the enrichment is otherwise similar. This resulted in identification of 3,827 and 1,674 methylated regions in the LNCaP prostate cancer cells and PrEC normal prostate cells respectively (see Additional File 5 for the top 300 regions identified in each cell type) with a false discovery rate of < 5% for both cell lines. Setting the smoothing parameter k to 5 or 9 and repeating the analyses showed that most of the candidate regions that were longer than 200 bp overlapped with those found by setting k = 7, with only 3.4% and 4.4% of all regions being new (i.e. not found when k = 7) for k = 5 or k = 9 respectively. Therefore, since changing k did not result in large differences, our choice of using k = 7 to correspond to the modal fragment size appears to be well justified. We also examined the effect of choosing different Z-score thresholds. The Z-score cutoff is essentially a tuning parameter that enabled a locus to be considered for enrichment. A larger Z cut-off for single probes requires a peak with higher amplititude. By lowering this cutoff, more candidate regions would be considered as potentially methylated. However, because an additive Z-statistic for the entire region is used to declare final enrichment, lowering the initial cutoff does not necessarily mean more enriched regions. One possibility is that adjacent regions are more likely to be merged into larger regions with a more permissive probe Z-score cutoff. By lowering the Z-score cut-off from 3 to 2.57 (representing the theoretical 99.5 percentile), we see that we only obtain a single new region that is longer than 200 bp with a final Z-score for the region greater than 4. Therefore, it appears that our choice of Z > 3 is a reasonable cut-off to use. It is likely that the smoothing parameter k and the Z-score threshold would need to be custom set for different enrichment based methylation tiling array applications, with the optimal values depending on the resolution of the microarray platform and the modal DNA fragment length.

We next carried out analyses to identify genes that were only methylated in the LNCaP cells and not in the PrEC cells, or vice versa. To do this, we merged overlapping methylated regions from each of these samples, and identified those merged regions that had an additive Z-score > 4 in on one sample and Z < 1 in the other sample, representing highly stringent criteria for differential methylation. This analysis resulted in identification of 163 regions in LNCaP cells that were not methylated in PrEC cells (see Additional File 6 for the top 50 hypermethylated regions in LNCaP vs. PrEC). Interestingly, we identified only 7 regions from Chromosomes 21 and 22 that were methylated in the PrEC cells that were not methylated in the LNCaP prostate cancer cells using our highly stringent criteria. This is in agreement with a previous report that used an independent method for identification of hypomethylated gene promoter CpG islands in prostate cell lines, in which the majority of hypomethylated promoter CpG islands arose from sex chromosomes and not from the autosomal chromosomes [23].

We next carried out extensive bisulfite sequencing experiments of regions that were identified as either methylated or unmethylated to assess the accuracy of the overall MBD-chip and analytical approaches. This included sequencing analysis of a total of 419 independent clones covering 22 genomic regions, spanning 446 CpG dinucleotides, for a total of ~5,800 methylation measurements at individual CpG dinucleotides. This analysis revealed correct classification of the methylation status of 19 out of the 22 tested regions, showing the high accuracy of our overall MBD-chip and analytical approaches (see Additional Files 2, 3 and 4). Several of these bisulfite-sequencing verifications will be discussed in more detail in subsequent subsections. In summary, the MBD-chip and associated analytical approaches can be used to accurately identify methylated regions in an absolute sense within a single sample and also to identify regions that are differentially methylated between samples.

We next assessed whether the identified methylated and differentially methylated regions had higher CpG dinucleotide content than would be expected if the regions were selected randomly from chromosomes 21 and 22. To do this, we first took the top 1200 identified methylated regions (top 600 from each chromosome) from each of the cell lines and created 500 simulated data sets that were matched to these regions in size and ensured that we only chose regions that had coverage on the tiling microarrays. We also created such simulated data sets for the top 50 (top 25 from each chromosome) regions that were identified as differentially methylated in the LNCaP cells compared to the PrEC cells. We could then compare the distribution of CpG dinucleotide content and number of regions overlapping CpG islands in these simulated data sets with these parameters in our actual observed data. This analysis showed that our identified methylated and differentially methylated regions, as expected, had significantly higher overlap with CpG islands and contained higher CpG dinucleotide content than the randomly selected simulated data sets (p << 0.002; see Additional File 7). These simulated data sets could also be used for assessing whether several other genomic annotations were enriched in our observed dataset compared to what would be expected by randomly choosing regions matched for appropriate parameters, as discussed in subsequent subsections.

### Enrichment of DNA methylation in intragenic or gene-proximal regions

We next carried out genomic annotation of the identified methylated and differentially methylated regions in the LNCaP and PrEC cells. For LNCaP and PrEC cells, 66 and 65 percent of methylated sequences respectively, were located within 3 kbp of known genes with only 34 and 35 percent of sequences lying in distal intergenic regions > 3 kbp of known genes (Table 1). There were no notable differences in region lengths between gene-associated and intergenic methylated regions within each cell line (Additional File 8). However, the average segment length of methylated regions in the LNCaP cells were significantly greater than those in the PrEC cells across all genome compartments examined. Examining regions that were hypermethylated in the LNCaP cancer cell line compared to the PrEC, 73 percent were located within 3 kbp of known genes.

**Table 1:** Characteristics of identified methylated and hypermethylated regions.

	Number of regions	Total length of regions (kbp)	% Gene upstream	% Gene downstream	% Exon	% Intron	% Intergenic
**LNCaP**	3827	1468.6	4.4	4.2	7.2	50.2	34
**PrEC**	1674	523.7	4.6	3	8.1	49.4	35
**Hypermethylated^1^**	163	147.5	9.5	5.4	4.2	54.4	26.6

^1^, Regions that are hypermethylated in the LNCaP cells compared to the PrEC cells.

**Figure 2:**
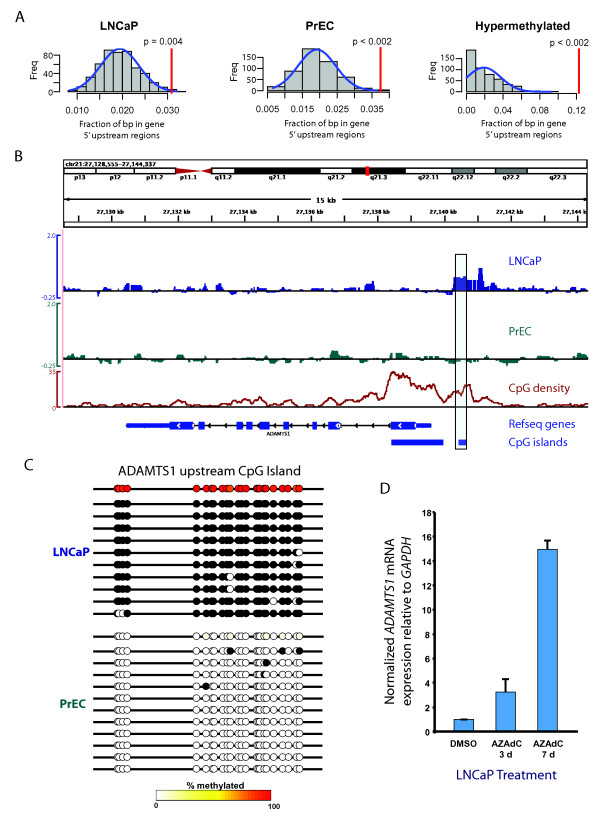
**Methylation of 5' gene upstream regions**. **A**, Methylated regions in LNCaP (*left*) and PrEC (*middle*) cells, and hypermethylated (*right*) regions in LNCaP vs. PrEC cells are significantly enriched within 2 kbp upstream of transcriptional start sites. The expected probability distribution for (hyper)methylated regions to overlap with 5' gene upstream regions is shown (gray bars and blue line). The red line indicates the observed fraction of base pairs overlapping 5' gene upstream regions in our actual dataset. **B**, DNA methylation signals (smoothed adjusted log_2_(M/T)) surrounding a representative 5' gene upstream region hypermethylated in LNCaP compared to PrEC. Annotations include chromosome coordinates (top), CpG density (number of CpGs in sliding 250 bp windows), Refseq genes, and CpG islands. The box indicates a region that was verified by bisulfite sequencing. **C**, Bisulfite verification of a hypermethylated region (boxed region from panel **(B)**) upstream of *ADAMTS1*. Circles represent position of CpGs. In the top line for each cell line the color of each circle represents the fraction of sequenced alleles that were methylated at that CpG according to the color scale (bottom). Each subsequent line represents the methylation pattern for each sequenced clone; black and white circles indicate methylated and unmethylated CpGs respectively. **D**, AZAdC induces re-expression of *ADAMTS1 *in LNCaP cells. Expression of *ADAMTS1 *with respect to that of *GAPDH *was measured by real time RT-PCR in LNCaP cells treated with vehicle (DMSO) or 1 μM AZAdC for 3 or 7 days.

Given the strong association of methylated regions with intragenic or gene proximal compartments, we more closely examined the localization of methylated regions within specific gene associated compartments. First, the LNCaP and PrEC cells showed significantly greater-than-expected enrichment for DNA methylation at 5' upstream regions of genes (Figure 2A). Additionally, this 5' upstream enrichment was even more pronounced in regions that were identified as hypermethylated in the LNCaP cells compared to the normal PrEC cells, consistent with the well-known trend for promoter hypermethylation in prostate cancer cells [4]. Figure 2B-C shows a representative hypermethylated 5' gene upstream region (*ADAMTS1 *gene) with the accompanying bisulfite sequencing validation. This 5' upstream methylation of the *ADAMTS1 *gene was associated with gene silencing since treatment of LNCaP cells with 5-aza-2'-deoxycytidine (AZAdC) resulted in ~15-fold induction of *ADAMTS1 *mRNA expression (Figure 2D).

**Figure 3:**
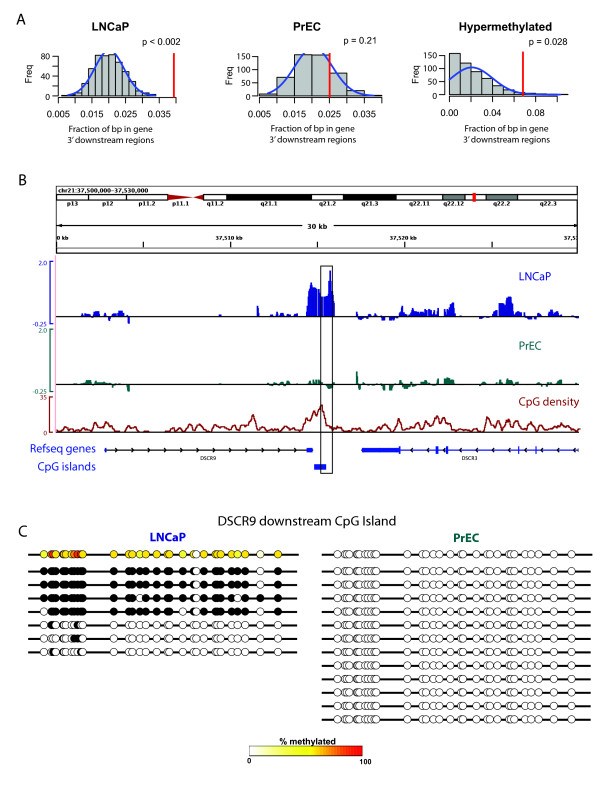
**Methylation of 3' downstream regions**. **A**, Methylated regions in LNCaP (*left*) and hypermethylated regions in LNCaP (*right*) are enriched for sequences within 2 kbp downstream of gene transcriptional termination sites. Conventions are the same as for Fig. 2A. **B**, DNA methylation signals at a representative 3' downstream gene hypermethylated region in LNCaP cells compared to the PrEC cells. Conventions are the same as for Figure. 2B. **C**, Bisulfite verification of a hypermethyalted region (boxed region from panel **(B)**) downstream of the *DSCR9 *and *DSCR3 *genes. Conventions are the same as for Figure. 2C.

Interestingly, DNA methylation was enriched at the 3' downstream regions of genes in the LNCaP cells but not the PrEC cells. Additionally, such 3' downstream regions were also enriched in regions that were hypermethylated in the LNCaP cells compared to the PrEC cells (Figure 3A). A representative hypermethylated 3' downstream gene region, in this case downstream of the *DSCR9 *gene, along with the bisulfite sequencing verification of this region, is shown in Figure 3B-C. We can speculate that such 3' downstream methylation may be involved in regulation of antisense transcripts [24], or in regulating transcriptional elongation or termination.

**Figure 4:**
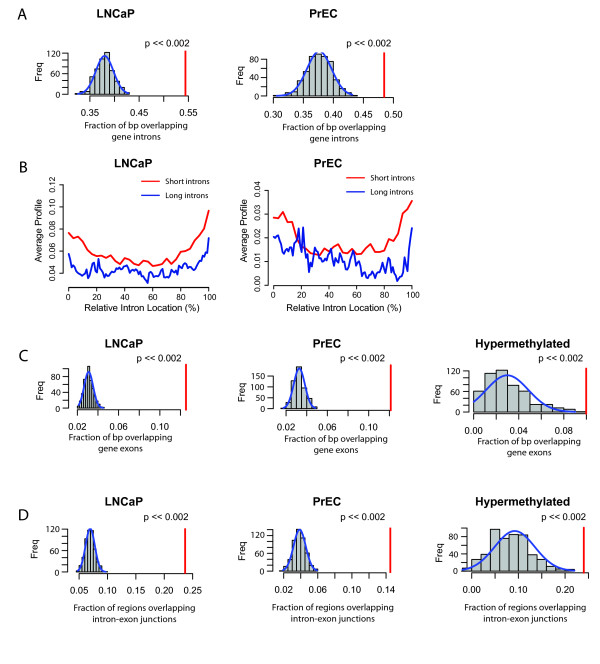
**Methylation of introns, exons and intron-exon junctions**. **A**, LNCaP (*left*) and PrEC (*right*) cells show significant enrichment for DNA methylation at intron sequences. Conventions are the same as for Figure. 2A. **B**, The average smoothed adjusted log_2_(M/T) across all short (842 - 2,715 bp) and long (2715 - 11,673 bp) introns was plotted with respect to the relative intron position (as a percentage of intron length), showing generally increased average signal towards the ends of introns in LNCaP (left) and PrEC (right) cells. **C - D**, Identified methylated regions in LNCaP (*left*) and PrEC (*middle*) cells, and hypermethylated (*right*) regions in LNCaP vs. PrEC cells, were highly enriched for overlap with exons and intron-exon junctions. Conventions are the same as for Figure 2A.

The majority of the gene-associated methylated regions and cancer differentially-methylated regions occurred in intron sequences. These intronic DNA methylation events were significantly enriched compared to what would be expected by random chance in our observed methylated region data sets for each of the cell lines (Figure 4A). To examine this enrichment more closely, all introns were scaled so that the position within each intron could be represented as a fraction between 0 and 100%, with 0% representing the start of the intron and 100% representing the end of the intron. The average smoothed adjusted log-ratio across all introns was plotted along this fractional position within the intron (Figure 4B). This analysis revealed a higher average DNA methylation signal towards the ends of introns compared to the middle of the introns, suggesting that much of the DNA methylation signals occurring in introns spanned intron-exon junctions. In further confirmation of this observation, we found that there was a significantly greater than expected enrichment of identified methylated and differentially methylated regions for overlapping with exon sequences and intron-exon boundaries specifically (Figure 4C-D). We can speculate that this DNA methylation and hypermethylation in cancer cells at intron-exon boundaries may be involved in suppression of alternative transcriptional start sites as has been reported recently [25]. Another potential role is in the regulation of splicing or exon usage. A recent report implicated specific histone modifications in regulating alternative splicing events [26]. We can speculate that these intron-exon DNA methylation events may also be involved in such regulation or in the establishment or reinforcement of these histone modifications.

### Methylated regions from intergenic sequences are highly enriched for conserved bases

**Figure 5:**
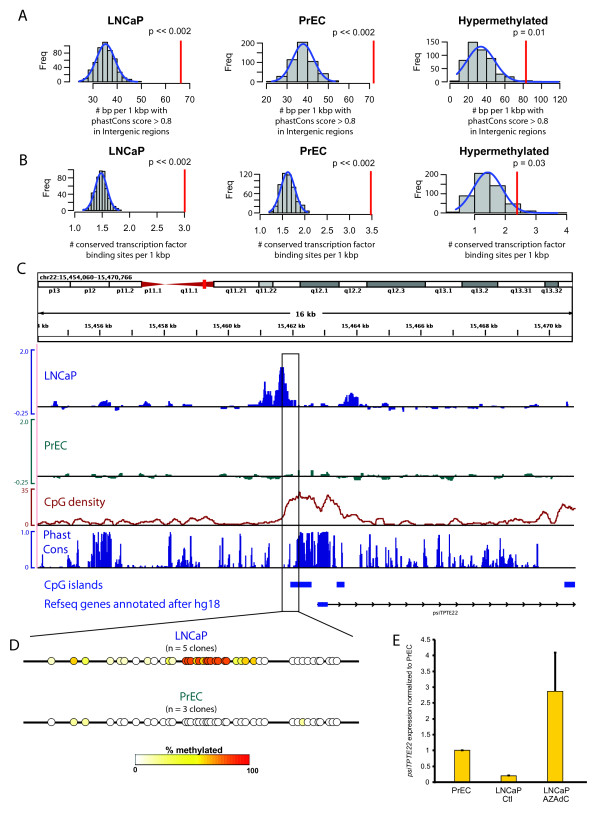
**Methylation of distal intergenic regions**. **A**, Methylated and hypermethylated distal intergenic regions were highly enriched for overlap with sequences with a high degree of conservation in mammalian and vertebrate species, as indicated by phastCons scores > 0.8. **B**, Conserved transcription factor binding sites are highly enriched in the methylated and hypermethylated regions. **A - B**, Conventions are the same as for Figure 2A. **C**, DNA methylation signals surrounding a hypermethylated region that was not near any known genes in the hg15 genome build. In addition to the annotations described in Figure 2B, the phastCons scores, representing the degree of conservation across 28 vertebrate and mammalian species, is shown. **D**, Bisulfite sequencing verification of the boxed region from **(C)**, following the same conventions at Figure 2C. Due to space limitations, only the summary schematics are shown. **E**, Normalized expression of *psiTPTE22*, a newly annotated pseudogene in hg18 that arises just downstream of the hypermethylated region shown in Figure 5C-D, in PrEC, DMSO (Ctl) and 1 μM AZAdC treated LNCaP cells.

Although the majority of methylated and differentially methylated regions were associated with intragenic or gene proximal regions, a significant fraction of identified regions occurred in distal intergenic sequences (see Table 1). There are at least two major hypotheses for methylation and differential methylation at these distal intergenic regions: i) these regions may not have any major regulatory role and may have become methylated spuriously with subsequent passenger maintenance of the methylation patterns, or ii) methylation/hypermethylation of these regions may have a role in physiological regulation of gene expression and/or carcinogenesis. In support of a regulatory role for several of these intergenic regions, we found that these regions exhibited significant enrichment for overlap with highly conserved sequences across many mammalian and vertebrate species (indicated by high phastCons scores > 0.8; [27]) compared with what would be expected by random chance (Figure 5A). Additionally, we found that the methylated and differentially methylated regions had a highly significant enrichment of conserved transcription factor binding sites (Figure 5B; [28]). Taken together, the overlap with high conservation and conserved transcription factor binding sites suggested that many of these regions that were not close to known genes may actually control transcription of unrecognized RNAs or distal genes.

To examine this more closely, we focused on one representative region in chromosome 22, which in our initial analyses using the UCSC hg15 Refseq genome annotations was identified as an intergenic region showing hypermethylation in the LNCaP cells compared to the PrEC cells in our microarray data (Figure 5C). This hypermethylation was verified by bisulfite sequencing experiments (Figure 5D). This region overlapped with an area of high conservation as denoted by high phastcons scores (Figure 5C). Interestingly, mapping this region to a more recent annotation of the human genome (UCSC hg18), we saw that a new pseudogene, called *psiTPTE22*, was mapped and annotated just downstream of this region (Figure 5C). A recent study also characterized the expression of a human endogenous retrovirus related gene (*psiTPTE22-HERV*) that mapped to this locus, and showed that this gene was likely silenced by DNA methylation of the upstream region in kidney tumors [29]. We next examined whether the differential methylation observed upstream of this pseudogene was involved in controlling expression of this pseudogene in the LNCaP and PrEC cells. Using primers specific for this gene, real time reverse transcriptase polymerase chain reaction (RT-PCR) analyses showed that PrEC cells, which lacked methylation of the upstream region, showed expression of *psiTPTE22*. In contrast, LNCaP cells, which showed a high degree of methylation of this region, showed significant underexpression of this gene compared to PrEC cells. However, treatment of LNCaP cells with AZAdC led to increased expression of *psiTPTE22 *(Figure 5E). We speculate that, like the methylation upstream of the *psiTPTE22/HERV *pseudogene, many of the other distal intergenic methylated and differentially methylated regions may also be involved in controlling transcription of previously unrecognized transcripts. Consistent with this, recent reports have suggested that up to 10-fold more genomic sequence may give rise to transcripts than is currently appreciated in genomic annotations [20].

If conserved intergenic regions showing hypermethylation in the LNCaP cells compared to PrEC cells have a regulatory role and can contribute to carcinogenesis, we would expect that such regions may show hypermethylation across a series of prostate cancer tissues compared to matched normal tissues. To test this, we selected a representative intergenic region that showed a high degree of conservation across vertebrate/mammalian species and for which we were able to readily design COMPARE-MS methylation assays. For this region, we assessed the degree of methylation in 21 subject-matched prostate tumor-normal pairs. Interestingly, this region showed consistent hypermethylation in the majority of the tumors compared to the matched normals, with a mean increase in each tumor compared to its matched normal of > 2-fold (p = 0.008; Additional File 9). The high frequency of hypermethylation of this region across the tumor-normal pairs suggests that such regions may be contributing to human prostate carcinogenesis. However, we would note that given the fact that there was some detectable methylation in the normal specimens for this particular intergenic region, we would not prioritize it highly for development as a biomarker for prostate cancer detection.

### Newly identified differentially methylated regions can serve as prostate cancer biomarkers

**Figure 6:**
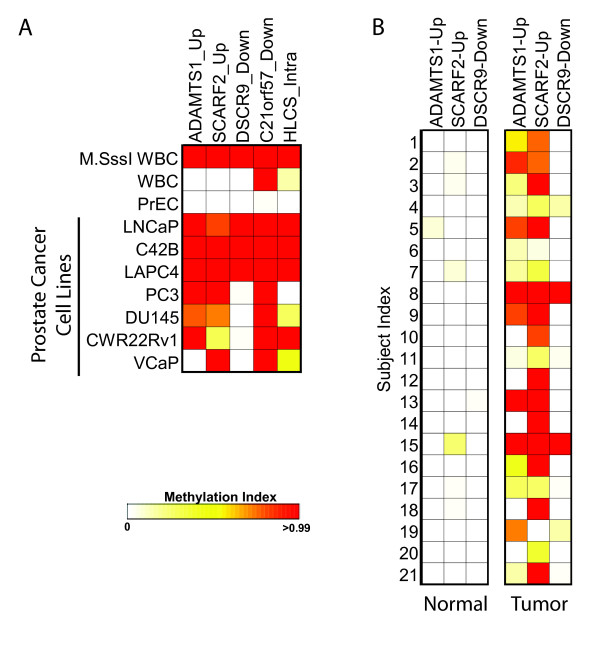
**Hypermethylated regions in LNCaP compared to PrEC cells can serve as biomarkers for prostate cancer**. **A**, DNA methylation at representative regions identified as hypermethylated in LNCaP cells compared to PrEC cells was measured in prostate cell lines using the COMPARE-MS assay as described previously [9]. The extent of methylation at each region is color scaled from white to red as shown, with white representing absence of detectable methylation, and red representing nearly complete methylation of all input copies. M.SssI-treated, completely-methylated, WBC DNA served as a positive control. All of the prostate cancer cell lines showed a high degree of methylation at multiple regions, while the PrEC normal prostate cells did not show any detectable methylation at these regions as expected. **B**, COMPARE-MS analysis of DNA methylation at three of the five regions from **(A) **showed significant methylation of at least one of the three regions in every tumor sample with very low or undetectable methylation in the matched normal tissue samples.

DNA methylation alterations have emerged as highly sensitive and specific biomarkers in many human cancers [30]. We assessed whether the differentially methylated regions identified in this study could be useful as DNA methylation biomarkers for effectively distinguishing prostate cancer from normal prostate. We selected representative regions that were differentially methylated between the LNCaP and PrEC cells, including two regions that were 5' upstream of known genes (*ADAMTS1 *and *SCARF2*), two regions that were 3' downstream of known genes (*DSCR9 *and *C21orf57*), and one region that was intragenic within a known gene (*HLCS*). Using the COMPARE-MS methylation detection assay [9], we confirmed that these regions were not methylated in the PrEC cells and were highly methylated in the LNCaP cells as predicted by our microarray and bisulfite sequencing analyses. Additionally, several other prostate cancer cell lines were methylated at these regions, with every prostate cancer cell line showing significant hypermethylation of at least three of these regions (Figure 6A). We next carried forward three of these regions (*ADAMTS1_Up*, *SCARF2_Up*, and *DSCR9_Down*) for testing in DNA isolated from tumor and paired adjacent normal tissues taken from men who underwent radical retropubic prostatectomy for management of primary prostate cancer (we omitted *C21orf57 *and *HLCS *associated regions because these showed some methylation in the WBC DNA and therefore may not serve as ideal biomarkers in human tissues, which may be heterogeneous in cell type; see Figure 6A). Interestingly, all three of these regions exhibited high frequency of methylation in the tumor samples (76%, 90.5%, and 19% for *ADAMTS1_Up*, *SCARF2_Up*, and *DSCR9_Down *respectively) with very infrequent methylation in the matched normal specimens (Figure 6B). Therefore, the regions that were identified to be differentially methylated in the LNCaP compared to PrEC cells as identified in this study by our overall MBD-chip and associated computational approaches are likely to be highly enriched for effective DNA methylation biomarkers for prostate cancer identification.

## Discussion

In this study, we have developed, refined and applied an MBD-chip approach along with accompanying computational analyses for comparison of chromosome-wide DNA methylation patterns in prostate cancer cells with those in normal prostate epithelial cells. We present several technological advances over previous affinity-enrichment based DNA methylation profiling approaches. First, the enrichment process has been streamlined and optimized for fairly small amounts of input DNA (only 300 ng of DNA were used for these studies). Second, compared to antibody based approaches which require the generation of single-stranded DNA for affinity enrichment of methylated DNA, the MBD-based enrichment approach offers the ability to enrich for methylated double-stranded DNA. Third, among the MBD-based approaches for affinity enrichment of methylated DNA, the fragment of the MBD2 protein used in this study is highly streamlined for binding methylated DNA with high affinity and selectivity [9]. The high selectivity of the MBD2-MBD polypeptide for methylated DNA and the high density of the oligonucleotide tiling microarrays covering all non-repetitive regions of chromosomes 21 and 22 with an average inter-probe spacing of ~10 bp allowed unbiased, high-resolution, chromosome-wide mapping of DNA methylation in the LNCaP prostate cancer cell line and the PrEC normal prostate epithelial cells in primary culture. Finally, we have developed novel computational approaches for analysis of affinity enrichment-based genome-wide DNA methylation data that correct for sequence bias in the methylation signal. The resulting methods greatly enhance the specificity and accuracy of the DNA methylation calls. These analytical methods were specifically optimized for interpretation of DNA methylation tiling microarray data. Knowing that DNA methylation occurs almost exclusively at CpG dinucleotides in adult somatic human cells, and that the MBD2-MBD polypeptide very selectively binds CpG methylated DNA, we were able to define a set of null probes that interrogate regions of the genome that contain an extremely low CpG density that should never be enriched. The signals arising from these probes allowed us to identify and correct for sequence biases that led to increased spurious signals in these regions. Additionally, one theoretical advantage of high-density tiling microarrays is that, if we assume independence between signals from adjacent probes, multiple consecutive probes exhibiting enrichment would multiplicatively increase our confidence that the overlying region was truly enriched. However, in many cases of tiling array data, the assumption of independence of adjacent probes is clearly not met and we therefore cannot easily calculate the confidence of signals arising in multiple consecutive or adjacent probes. In our own data also, we saw that the raw smoothed log-ratios from null probes were highly autocorrelated with the smoothed log-ratios from adjacent probes. However, correcting for the GC content sequence biases using the null probes eliminated this autocorrelation, allowing us to assume independence in signals arising from consecutive null probes. The resulting analyses were highly accurate for absolute methylation calls, with false discovery rates of < 5% and concordance with bisulfite sequencing data of ~90%.

In this study, we restricted analysis to absolute (qualitative) DNA methylation calls because significant new computational methods development is necessary for quantitative analysis of DNA methylation from affinity-enrichment based genome-wide DNA methylation data. This is because deriving quantitative information regarding the fraction of input DNA that is methylated at a given locus from affinity-enrichment based approaches is confounded by multiple issues that are independent of the fraction of methylated input DNA fragments. First, the degree of enrichment is clearly influenced by the density of methylated CpGs around a given locus, and this appears to show a non-linear dependence. Second, the degree of enrichment is likely influenced by various sequence effects and biases. These biases we have in large part been able to isolate and adjust for in qualitative analyses (as described in the manuscript), but significant further research is required to understand how such parameters influence the ability to quantitate methylation levels at a given locus in a specific sample. Third, the degree of enrichment at a given locus is influenced by the total amount of captured species in a given sample. That is, because the same amount of total DNA is hybridized (or sequenced) for each sample, the degree of signal at a given region is influenced both by the amount of methylation at that region and by the total number of methylated molecules making up the enriched sample. Unfortunately, it seems likely that each of these parameters can influence the other parameters in a non-linear and currently unpredictable fashion. In ongoing studies, we are developing methodologies to overcome these issues in order to facilitate accurate quantitative estimates of DNA methylation from enrichment-based genome-wide DNA-methylation data. In the meanwhile, our accurate approaches for qualitative assessment of DNA methylation have allowed significant new biological insights into the differences in chromosome-wide DNA methylation patterns in a cancer/normal model system.

In the classically held view, DNA methylation patterns in cancer cells differ from normal cells in at least two major ways [31,32]. First, they often harbor hypomethylation of repetitive elements and of regions of the genome with low CpG density. Our methods did not directly interrogate this aspect of DNA methylation biology since repetitive elements were excluded from the arrays to avoid cross-hybridization signals and because our method, like other restriction enzyme and enrichment based genome-wide DNA methylation assays, cannot robustly detect differential methylation in regions with very low CpG density [5,6]. Second, cancer cells are thought to become hypermethylated mostly in CpG islands at the promoters of genes, resulting in epigenetic silencing of those genes. Accordingly, the majority of genome-wide DNA methylation assays have focused on CpG islands and promoters using various types of microarray formats with probes that selectively interrogate such regions. Here, we assessed whether DNA hypermethylation changes in cancer cells occur mostly in gene promoter CpG islands by carrying out an unbiased assessment of DNA methylation across all non-repetitive regions of chromosomes 21 and 22 (without bias to promoters, genes, or other annotations) in prostate cancer and normal prostate cells.

Annotation of the identified methylated regions revealed a significant clustering of DNA methylation in gene-associated compartments of the genome in both the cancer and normal cells, and in regions found to be hypermethylated in the cancer cells. We identified numerous 5' gene upstream regions that were methylated in the cancer and normal cells, some of which were differentially methylated in the cancer cells. For some of these regions, we confirmed that demethylation using a methyltranferase inhibitor led to re-expression of the associated gene, suggesting that methylation of these regions was indeed involved in epigenetic silencing of the associated gene. Two of these regions were confirmed to be novel biomarkers for prostate cancer in an independent set of prostate cancer cell lines and prostate cancer tissues.

Interestingly, we also found significant enrichment for methylation greater than would be expected by random chance for several other gene-associated genome compartments. For instance, we found that methylation of 3' gene downstream regions was enriched to nearly the same extent as 5' gene upstream regions in the LNCaP prostate cancer but not PrEC normal prostate cells, and was also enriched in the cancer hypermethylated regions. Recent reports have suggested that many genes may have antisense transcripts that may be involved in the regulation of the sense transcripts [24]. We speculate that methylation of the 3' downstream regions may be involved in the regulation of such antisense transcripts. Another possibility is that methylation of such regions is involved in regulating transcriptional elongation/termination or transcript processing such as polyadenylation. Further studies will be required to understand the role of the 3' gene downstream methylation events.

Introns and exons also showed significant enrichment of methylation in the cancer and normal cells. Interestingly, exon sequences and intron-exon junctions showed an extremely high degree of enrichment within methylated regions in cancer and normal cells, as well as in hypermethylated regions in the cancer cells. Luco et al., recently showed that histone methylation patterns occurring at intron-exon boundaries can play a role in regulating alternative splicing of mRNA [26]. We speculate that DNA methylation patterns may help to reinforce these histone methylation patterns or may also be directly involved in regulation of alternative splicing. Another recent report has suggested that DNA methylation patterns occurring within gene bodies may be involved in regulation of alternative transcriptional start sites [25]. To our knowledge, neither of these or other previous reports compared gene body methylation in cancer and normal cells. Our data suggest that such gene body DNA methylation can become abnormally increased in prostate cancer cells. We can speculate that cancer cells can take advantage of this regulatory machinery to activate oncogenes or silence tumor suppressors by dysregulating production of alternative transcripts and spliceoforms.

Although the majority of methylated regions overlapped with gene-associated genome compartments, a significant fraction of regions (~30 - 40%) were distal intergenic, occurring at least 3 kbp away from any known genes. Several such distal intergenic regions showed hypermethylation in the cancer cells compared to the normal cells. Interestingly, these intergenic methylated and cancer hypermethylated regions were significantly enriched for a high degree of conservation across several mammalian and vertebrate species, suggesting that there are significant evolutionary pressures against changes at these regions. We can speculate that these regions are involved in long range regulation of genes. Another possibility is that some subset of these intergenic methylated regions are involved in regulation of nearby transcripts that are not yet annotated or known. Consistent with both of these hypotheses, the genomic methylated regions are highly enriched for conserved transcription factor binding sites.

Regardless of the function of the cancer hypermethylated regions, it is apparent that many of these have significant potential in serving as DNA methylation biomarkers of prostate cancer. Cancer hypermethylated regions from different annotation categories (5' gene upstream, 3' gene downstream, intergenic) were frequently methylated in prostate cancer cell lines but not the normal prostate epithelial cells. A few of these (regions associated with *ADAMTS1, SCARF2*, and *DSCR9*) were tested further, and in combination, showed ~100% sensitivity and ~85% specificity for prostate cancer compared to matched adjacent benign tissues.

We envision several possibilities for application of the methodologies presented here for cancer biomarker development. For example, the MBD-enrichment based genome-wide DNA methylation approaches can be applied to tumor-normal pairs from several subjects of a given cancer type to assess whether there are any high-frequency DNA methylation changes that can distinguish tumor vs. normal tissue. Then, sensitive DNA methylation analytical techniques, such as COMPARE-MS [9], real-time MSP [19] or MethyLight [33], Methyl-BEAMing [34], etc., can be used to measure a panel of these DNA methylation alterations in blood, urine, stool, biopsies or other patient biomaterials. A different strategy, analogous to one that was recently described [35], would involve development of personalized DNA methylation biomarkers. In this strategy, for a given individual, technologies similar to those presented here would be applied to profile the genome-wide DNA methylation patterns distinguishing the individual's tumor from their own normal tissues. These personalized methylation alterations could then be followed in blood, urine or other biospecimens using the various sensitive DNA methylation techniques listed above to track response to therapy, follow disease burden, etc. Of course, such strategies will require significant testing prior to clinical implementation.

The overall MBD-chip approach described here should be broadly applicable to characterizing genome-wide DNA methylation patterns and to identify novel DNA methylation biomarkers for various diseases. The MBD2-MBD polypeptide is now commercially available as part of kits for enriching methylated DNA marketed by different companies (e.g. ClonTech, Invitrogen), and is therefore easily accessible to the research community. Additionally, tiling microarrays interrogating all non-repetitive regions of the entire genome of multiple species, including humans, are now available through various companies including Affymetrix, Nimblegen, and Agilent. Therefore, the methodologies presented here can be readily applied to analysis of the entire human genome. Furthermore, these methods should be easily adaptable to analysis with next generation sequencing [17]. For instance, recent studies have demonstrated that next generation sequencing platforms also produce significant sequence biases in data produced by their applications [36], including DNA methylation data [18]. It has been shown that sequence biases and amplification bias can affect affinity-enrichment based DNA methylation data produced by next generation sequencing platforms [18]. The general principle of using regions of the genome with ultra-low CpG content to correct such artifactual effects in DNA methylation data introduced by technology platforms should be generally applicable. Methods such as those presented here are poised to facilitate the thorough examination of DNA methylation patterns genome-wide in health and disease.

## Conclusions

We have developed and refined MBD-Chip and associated computational methods for analysis of DNA methylation using high-resolution oligonucleotide tiling microarrays. These analyses were deployed to compare chromosome-wide DNA methylation patterns in normal and malignant prostate cells, revealing significant enrichment of DNA methylation and hypermethylation of gene-proximal genomic regions, including 5'-gene upstream regions, 3'-gene downstream regions, and those spanning intron-exon junctions. Interestingly, intergenic methylated and hypermethylated regions showed a significant enrichment for harboring highly conserved sequences across vertebrate species. Several of these newly identified cancer hypermethylated regions were highly effective as DNA methylation based biomarkers capable of sensitively and specifically distinguishing malignant from normal prostate tissues and cell lines.

## Methods

### Cell lines, tissue specimens, treatments, and DNA/RNA extraction

LNCaP and PrEC cells were grown and maintained as described previously [19,23]. Fresh frozen blocks of tumor and matched non-cancer containing tissues from men that underwent radical prostatectomy for treatment of clinically localized adenocarcinoma of the prostate were obtained from the Brady Urological Institute Prostate Specimen Repository at Johns Hopkins. These specimens ranged in Gleason score from 6 - 9, and the pathological stage ranged from T2N0Mx to T3bN0Mx. Microscopic tissue sections were stained by hematoxylin and eosin (H&E) and examined to ensure purity of tumor-containing and non-tumor containing regions. Purity of tumor samples was estimated to be between 70-90% pure. Subsequent tissue sections were taken for DNA isolation, and then additional H&E sections were examined to ensure continuity of the diagnoses. Genomic DNA was isolated from tissue specimens and cells using the DNeasy kit (Qiagen) according to the manufacturer's protocols. WBC DNA was purchased from Novagen. Treatment of DNA samples with M.HhaI and/or M.HpaII, and M.SssI DNA methyltransferases (NEB) were carried out according to the manufacturer's recommended protocol. For DNA demethylation and gene re-expression studies, LNCaP cells were treated every day for up to one week with 1 μM AZAdC in DMSO or, as a control, with an equivalent volume of DMSO carrier. Cells were harvested by trypsinization at 3 and 7 days and total RNA was isolated using the RNeasy kit (Qiagen) according to the manufacturer's protocols.

### MBD-Chip sample preparation

300 ng of genomic DNA samples were separated into three fractions and each fraction was digested in a 10 μL reaction with either R.AluI (NEB), R.HaeIII (NEB), or R.MseI (NEB) according to the manufacturer's recommendations. Splitting samples into three reactions and digesting each reaction with one of three 4-base recognition sequence restriction enzymes allowed fragmentation of DNA while maintaining high representation of genome sequences in fragments > 100 bp. The three restriction digestion reactions were then pooled to reconstitute the original sample. Half of this digested sample was set aside and designated as "input control". The remaining half was designated as the "unknown methylated fraction".

The "unknown methylated fraction" was subjected to enrichment for densely methylated DNA sequences by capture with MBD2-MBD immobilized on magnetic beads using a procedure similar to that used in COMPARE-MS as described previously [9]. Briefly, in a pre-capture step, 2.5 μL of Protein G Magnetic Beads (NEB, Beverly, MA) were gently shaken for 1 hour at room temperature with 1 μg of PentaHis Antibody (Qiagen, Valencia, CA), 160 nM MBD2-MBD-6His, and 200 ng of an unmethylated self-ligated TOPO-TA plasmid (Invitrogen, Carlsbad, CA), in 97.5 μL of BW Buffer(4% glycerol, 1 mM MgCl_2_, 0.5 mM EDTA, 0.5 mM DTT, 50 mM NaCl, 10 mM Tris-HCl (pH 7.4), 0.2% Tween-20, and 1X Complete EDTA-free Protease Inhibitor cocktail). Unbound antibody and MBD polypeptides were removed by immobilizing beads on a Magnetight HT96 magnetic rack (Novagen, San Diego, CA) and removing the supernatant. The methylated fraction samples were diluted in 100 μL of BW buffer and then incubated with the protein G magnetic beads for 3 hours at room temperature with gentle shaking. The beads were then immobilized on the Magnetight HT96 rack and washed five times with BW Buffer. After the final wash, the bound methylated fraction DNA was eluted by adding 20 μL of 1 mM Tris-HCl pH 8.0 and heating to 95°C for 15 minutes. The magnetic beads were again immobilized on the Magnetight HT96 rack and the supernatant containing the eluted methylated fraction DNA was removed and stored until unbiased amplification. Purified recombinant MBD2-MBD polypeptides were produced as described previously [9].

The input controls and unknown reactions were subjected to unbiased, whole-genome amplification via a random-primed amplification (R-PCR) strategy as described previously [37-40]. Briefly, DNA was subjected to two successive one strand synthesis reactions using Sequenase DNA polymerase (USB) and Primer A (5'-GTTTCCCAGTCACGATCNNNNNNNNN), featuring a degenerate 3' end for random-primed polymerization and a specific 5' sequence. This reaction was then subjected to 25 cycles of PCR amplification with Primer B (5'-GTTTCCCAGTCACGATC). After verification of robust amplification by 1% agarose gel electropheresis, amplified products were purified with the Qiagen PCR purification kit and quantified by standard UV absorbance spectrometry.

Whole genome amplified DNA was then subjected to partial digestion for 6 min at 37°C with 5 Units of DNAse I (Epicentre) in a 40 μL reaction containing 1x One-Phor-All buffer (GE Healthcare). Fragmented DNA was 3'-biotin end-labeled by incubation with 70 μM biotin-ddATP, 100 Units Terminal DNA Transferase (TdT; Roche), 2.5 mM CoCl_2_, and 1x TdT buffer (Roche) in a 70 μL reaction. Labeled DNA was hybridized according to the manufacturer's instructions, to GeneChip^® ^Human Chromosome 21/22 Tiling 1.0R Array Sets (Affymetrix) consisting of 3 microarrays containing 25-mer oligonucleotide probes that are tiled across all non-repetitive genomic sequences on chromosomes 21 and 22 with 35 bp resolution (i.e. average distance between probes is 10 bp). Hybridization reactions and scanning were carried out by the Johns Hopkins Microarray Core facility. Each sample was analyzed in duplicate experiments.

### Pre-processing and analysis of microarray data

Microarray data pre-processing and analysis were carried out using R statistical programming language (R Development Core Team, http://www.r-project.org) and Bioconductor software [41], except where noted. Microarray probe sequences were mapped to the hg15 and hg18 UCSC genome builds. A set of "null probes" was defined as those probes that mapped to three different long regions of chromosomes 21 and 22 with very low CpG content of < 0.4%. For each sample, the mean log_2 _intensity on each probe between the duplicate experiments was formed to calculate the log_2 _ratio between the methylated (M) and the total input (T) fractions. The probe effect of the log_2 _ratio was estimated from null probes stratified by probe GC content as the median log ratio for each GC stratum. This estimated probe effect was subtracted from the raw log-ratio for all probes to form the adjusted log ratio. A running median of this adjusted log_2_(M/T) ratio was calculated across a sliding window of 7 adjacent probes and was taken as the final pre-processed measure of methylation. For each probe, a Z-score, calculated as the number of standard deviations from the median log_2_(M/T) of the null probe bin with the same GC content, was determined. All probes with Z-score > 3 were considered as potentially enriched. All enriched probes within 250 bp of proximity were merged to form enriched genomic segments and the sum of Z-scores in the segment was calculated. All genomic segments with Z > 4 were considered as significantly methylated. Since the smoothed adjusted log_2_(M/T) in the null regions were found to be approximately normally distributed, this Z-score threshold corresponds to p < 3.2E-5. To estimate false discovery rates, this analysis was performed on replicate total input fractions in which the log_2_(Total Input*_replicate1_*/Total Input*_replicate2_*) was used in place of log_2_(M/T). The number of regions with Z > 4 in this absolute null dataset was used to estimate the false discovery rate for each sample. To identify differentially methylated regions that were likely to be methylated in one of the cell lines and show absence of methylation in the other, we considered all regions that were considered to be methylated in the LNCaP or PrEC samples and merged overlapping regions together. For these regions, we used the following highly stringent criteria for identification of differentially methylated regions: i) Z > 4 for the region in either the LNCaP or PrEC sample; ii) Z < 1 in the other sample; iii) region length > 500 bp.

### Analysis of whether methylated regions were enriched for overlap with various genomic sequence annotations

CEAS software [42,43] was used to calculate the fraction of identified methylated and differentially methylated regions overlapping with various genome annotations (introns, exons, 5' gene upstream, 3' gene downstream, distal intergenic) and to carry out average profile analysis of smoothed adjusted log_2_(M/T) values across all short (842 - 2,715 bp) and long (2715 - 11,673 bp) intron sequences represented on the microarrays. For analyses examining whether methylated and differentially methylated regions were enriched for various genome annotations (CpG dinucleotide content, overlap with CpG islands, bp overlapping 5' gene upstream regions, 3' gene downstream regions, intron regions, exon regions, intron-exon junctions, conserved intergenic sequences with phastCons scores > 0.8, and overlap with conserved transcription factor binding sites) we used annotations publicly available through the UCSC genome browser database [44] or the NCBI ftp server (ftp://ftp.ncbi.nih.gov/genomes/MapView). We then took the top 1200 regions (top 600 from each of chr 21 and 22) for the LNCaP and PrEC samples, and the top 50 differentially methylated regions (top 25 from each of chr 21 and 22), and used a custom Java (Sun Corporation) program to generate M = 500 *in silico *data sets in which we randomly chose regions that were matched to the regions in our experimental data set for length and coverage on the microarrays. Randomly chosen regions with the same length as the experimental data set were generated in rank order (ranked by the standardized Z-statistic). Start sites for randomly selected regions were constrained to start sites of probes represented on the microarrays to control for bias introduced by probe design/selection on the microarrays. For each *in slico *simulated dataset, region selections were constrained to not overlap with previously selected regions. Each of these datasets were then annotated for overlap with the same annotations listed above. For each type of annotation, we calculated the probability that the experimental dataset was enriched for that type of annotation compared to random chance as,

where N_{random > experimental} _is the number of random datasets with annotation measurement greater than that in the experimental data set, and Q is the total number of random datasets generated.

### Bisulfite sequencing

Bisulfite sequencing was carried out as described previously [23]. Primers and associated annealing temperatures are shown in Additional File 10.

### Compare-MS DNA methylation analysis

For COMPARE-MS analysis of DNA methylation at newly identified cancer hypermethylated regions in prostate cancer cell lines, tumor-normal paired tissues, and reference samples, DNA samples were digested with R.AluI and R.HhaI (NEB) and methylated fragments were enriched and analyzed by real-time PCR as described previously [9]. COMPARE-MS primers and corresponding annealing temperatures for real time PCR are shown in Additional File 10.

### Quantitative RT-PCR

Quantitative RT-PCR analysis of *ADAMTS1 *and *GAPDH *gene expression was carried out using Taqman assays (Applied Biosystems) with procedures for reverse transcription and real-time PCR as described previously [45]. For the *psiTPTE22 *gene, SYBR green based real-time RT-PCR was carried out using 400 nM forward (5'-GTATGCTCTGACAACTATGAC) and reverse (5'-GAGAGTGACATCCAGTAAGAC) primers, in 25 μL reactions containing 1x SYBR Green RT-PCR reaction mix (Biorad), 0.5 μL of iScript reverse transcriptase (Biorad), and 50 ng total RNA. Cycling conditions were 55°C for 30 min, 95°C for 3 min, followed by 40 cycles of 95°C for 30 sec, 58°C for 30 sec, and 72°C for 30 sec. All real-time PCR analyses were performed on Biorad iCycler thermal cyclers.

## Abbreviations

MBD: Methyl-binding domain polypeptide; WBC: White blood cells; R-PCR: Random-primed polymerase chain reaction; ChIP-Chip: Chromatin immunoprecipitation microarray; MAT: Model-based analysis of tiling arrays; GC: Guanine and cytosine; Chr: Chromosome; Bp: Base pairs; Kbp: Kilobase pairs; AZAdC: 5-aza-2'-deoxycytidine; RT-PCR: Reverse transcriptase polymerase chain reaction; H&E: Hematoxylin and eosin

## Competing interests

S.Y., A.M.D., and W.G.N. are co-inventors of intellectual property (United States Patent Application No. 60/775,980) entitled, "COMPARE-MS: Method for rapid, sensitive and accurate measurement of DNA methylation."

## Authors' contributions

S.Y. designed and coordinated the study, performed experiments, performed data analysis and interpretation, and wrote the manuscript. Z.W., M.J.A., J.D.M., B.C., S.Y., and R.A.I., developed and performed computational methods and carried out data analysis. M.C.H., D.E., R.B., L.H., and Q.Z. performed experiments and assisted in data analysis. A.M.D. carried out pathological analysis of prostate tissues and assisted in study design. Z.W., M.C.H., M.J.A, A.M.D., and W.G.N, assisted in writing the manuscript. W.G.N. participated in the design and coordination of the study. All authors read and approved the final manuscript.

## Supplementary Material

Additional file 1 **Pre-processing of MBD-chip data by correcting for GC content-based probe-fraction interaction effects**. **A**, The log_2_-ratio of intensity from the methylated fraction to the total input (log_2_(M/T)) in null probes (probes interrogating regions of chr 21 and 22 with very low CpG density of <5 per 10 kbp) increases as a function of increasing probe G+C content. **B**, The unadjusted log_2_(M/T) shows a strong autocorrelation (left). Adjusting for G+C content nearly eliminates any significant autocorrelation. **C**, A quantile-quantile (Q-Q) plot of observed quantiles of the running median (smoothed) of adjusted log_2_(M/T) to theoretical quantiles derived from a standard normal distribution, shows that the smoothed adjusted log_2_(M/T) highly resembles what would be expected for a running median of a standard normal distribution (red diagonal line). Click here for file

Additional file 2 **Representative results and validation of the MBD-chip pre-processing approach**. **A**, The raw log_2_(M/T), smoothed log_2_(M/T), and the smoothed adjusted log_2_(M/T) are shown for LNCaP cells for a representative 15 kbp region on chr 22. The raw and smoothed log_2_(M/T) appear to be high throughout the region. A running median of the adjusted log_2_(M/T) attenuates the signal in most regions (e.g., boxed region on the left) but maintains a high signal in a region upstream of the *ADAMTS1 *gene (boxed region on the right). The shown region is annotated with the chromosome coordinates (top), Refseq genes, and CpG islands. **B**, Representative results of bisulfite sequencing experiments verifying the accuracy of the smoothed adjusted log_2_(M/T) as a measure of DNA methylation. Note that the boxed region on the left, which has very low log_2_(M/T) signals from the microarrays (panel (A)), shows near absence of methylation of the underlying CpG island, while the boxed region on the right, which shows a relatively high log_2_(M/T) signal from the microarrays (panel (A)), shows nearly complete methylation of the underlying CpG island. Circles represent positions of CpGs. In the top lines for each region, the color of each circle represents the fraction of sequenced alleles that were methylated at that CpG according to the color scale. Each subsequent line represents the methylation pattern for each sequenced clone; black and white circles indicate methylated and unmethylated CpGs respectively. This convention is used for all subsequent bisulfite sequencing figures. Click here for file

Additional file 3 **Bisulfite sequencing verification data of methylated regions identified by MBD-chip in the LNCaP and PrEC samples**. The "BSF data" columns show results from bisulfite sequencing of an amplicon (chromosomal coordinates of each bisulfite sequencing amplicon are shown above each region) within the region called by the MBD-chip analysis. For each methylated region identified by the MBD-chip analysis, the cell line, chromosome coordinates, and additive standardized Z-score for each region are listed in the columns labeled "MBD-chip data". Conventions for bisulfite sequencing are the same as those for Additional File 2 panel B. Click here for file

Additional file 4 **Bisulfite sequencing verification data of regions that were identified as hypermethylated in the LNCaP compared to the PrEC cells**. Conventions are the same as those for Additional File 3. Click here for file

Additional file 5 **Top 150 methylated regions from each of chromosomes 21 and 22 in LNCaP prostate cancer cells and PrEC normal prostate epithelial cells**. Click here for file

Additional file 6 **Top 25 regions from each of chromosomes 21 and 22 that are hypermethylated in LNCaP vs. PrEC**. Click here for file

Additional file 7 **Identified methylated and hypermethylated regions show a much higher CpG content and overlap with CpG islands than would be expected by random chance**. In each panel, the distribution plot shows the expected probability (y-axis) due to random chance of identifying regions with the indicated average fraction of regions overlapping with CpG islands (left panels) or the indicated number of CpGs per 1 kbp (right panels) as plotted on the x-axis. The gray bars represent a non-parametric distribution for the expected probabilities. The overlying blue line represents a best-fit normal distribution of the expected probabilities. The vertical red line indicates actual observed data. Click here for file

Additional file 8 **Segment lengths of methylated regions in LNCaP cells are significantly longer than those of PrEC cells, but do not differ significantly across different genome compartments within each cell line. **Shown are box-and-whisker plots representing the distribution of segment lengths of methylated regions. The box represents the 25^th ^to 75^th ^percentile, and the whiskers represent the 5^th ^and 95^th ^percentiles. Red symbols indicate outliers. Click here for file

Additional file 9 **Frequent hypermethylation of a representative conserved intergenic region**. **A**, DNA methylation signals (smoothed adjusted log_2_(M/T)) surrounding a representative intergenic region that was identified to be hypermethylated in the LNCaP cells compared to PrEC cells. The shown region is annotated with the chromosome coordinates (top), CpG density (number of CpGs in sliding 250 bp windows), and PhastCons scores. The boxed area represents the region identified to be hypermethylated. Note that the region overlaps sequences with high conservation as indicated by high PhastCons scores. **B**, A waterfall plot of the extent of hypermethylation of the boxed region from panel (A) in paired tumor-normal prostate tissues. Click here for file

Additional file 10 Primers for bisulfite genomic sequencing, COMPARE-MS, and analysis of MBD2-MBD enrichment. Click here for file
