# Supplementary figures and images for: Chromosome-wide mapping of DNA methylation patterns in normal and malignant prostate cells reveals pervasive methylation of gene-associated and conserved intergenic sequences
Source: BMC Genomics. 2011 Jun 13;12:313. doi: 10.1186/1471-2164-12-313 (PMC3124442; doi:10.1186/1471-2164-12-313)

Additional file 1.

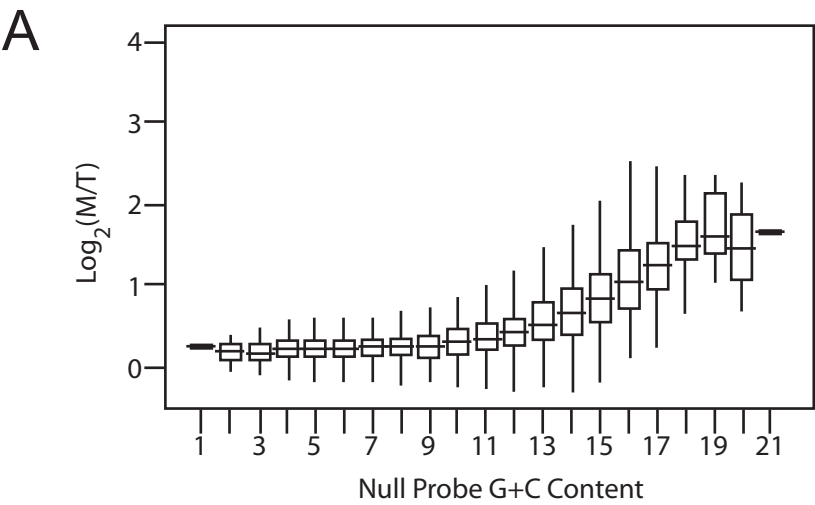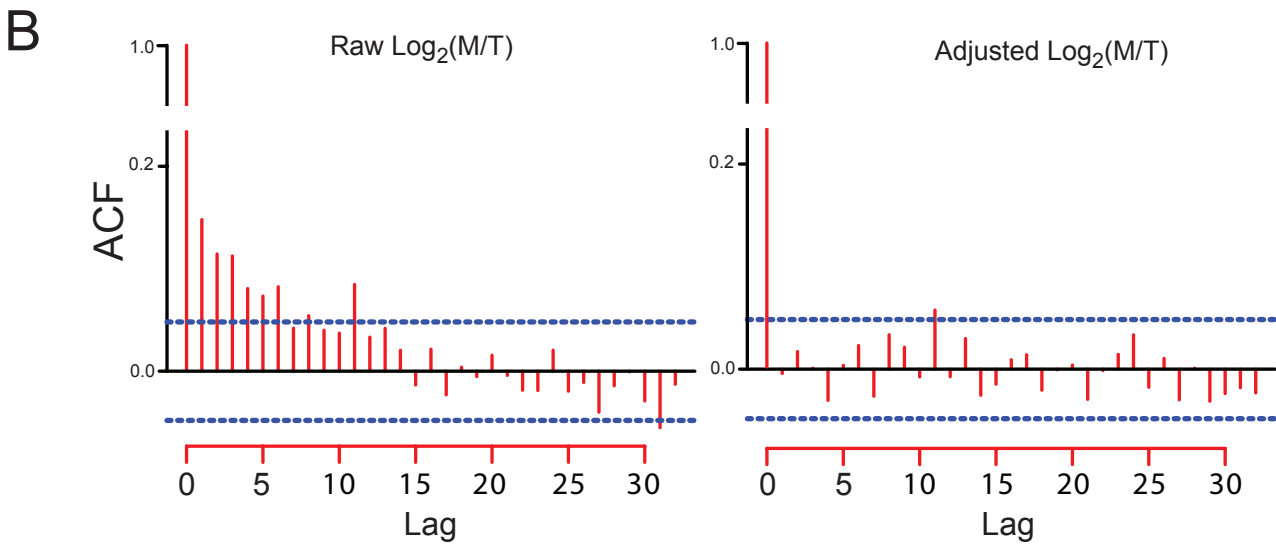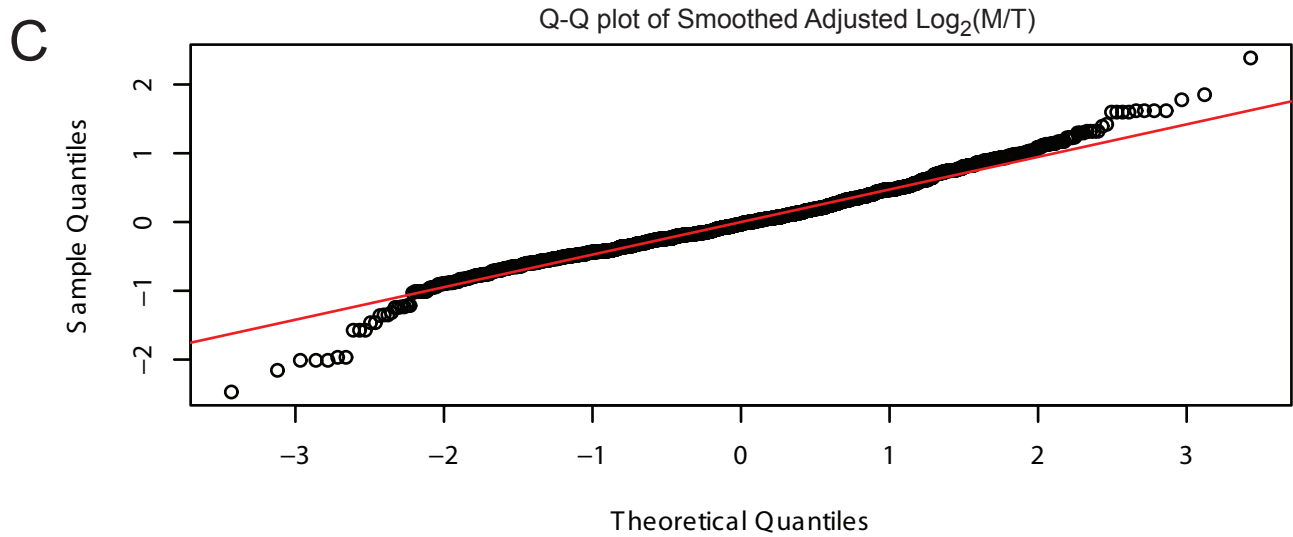

Supplement: Additional file 1 — Pre-processing of MBD-chip data by correcting for GC content-based probe-fraction interaction effects. A, The log2-ratio of intensity from the methylated fraction to the total input (log2(M/T)) in null probes (probes interrogating regions of chr 21 and 22 with very low CpG density of <5 per 10 kbp) increases as a function of increasing probe G+C content. B, The unadjusted log2(M/T) shows a strong autocorrelation (left). Adjusting for G+C content nearly eliminates any significant autocorrelation. C, A quantile-quantile (Q-Q) plot of observed quantiles of the running median (smoothed) of adjusted log2(M/T) to theoretical quantiles derived from a standard normal distribution, shows that the smoothed adjusted log2(M/T) highly resembles what would be expected for a running median of a standard normal distribution (red diagonal line). [file 1471-2164-12-313-S1.PDF]

Additional file 2.

A

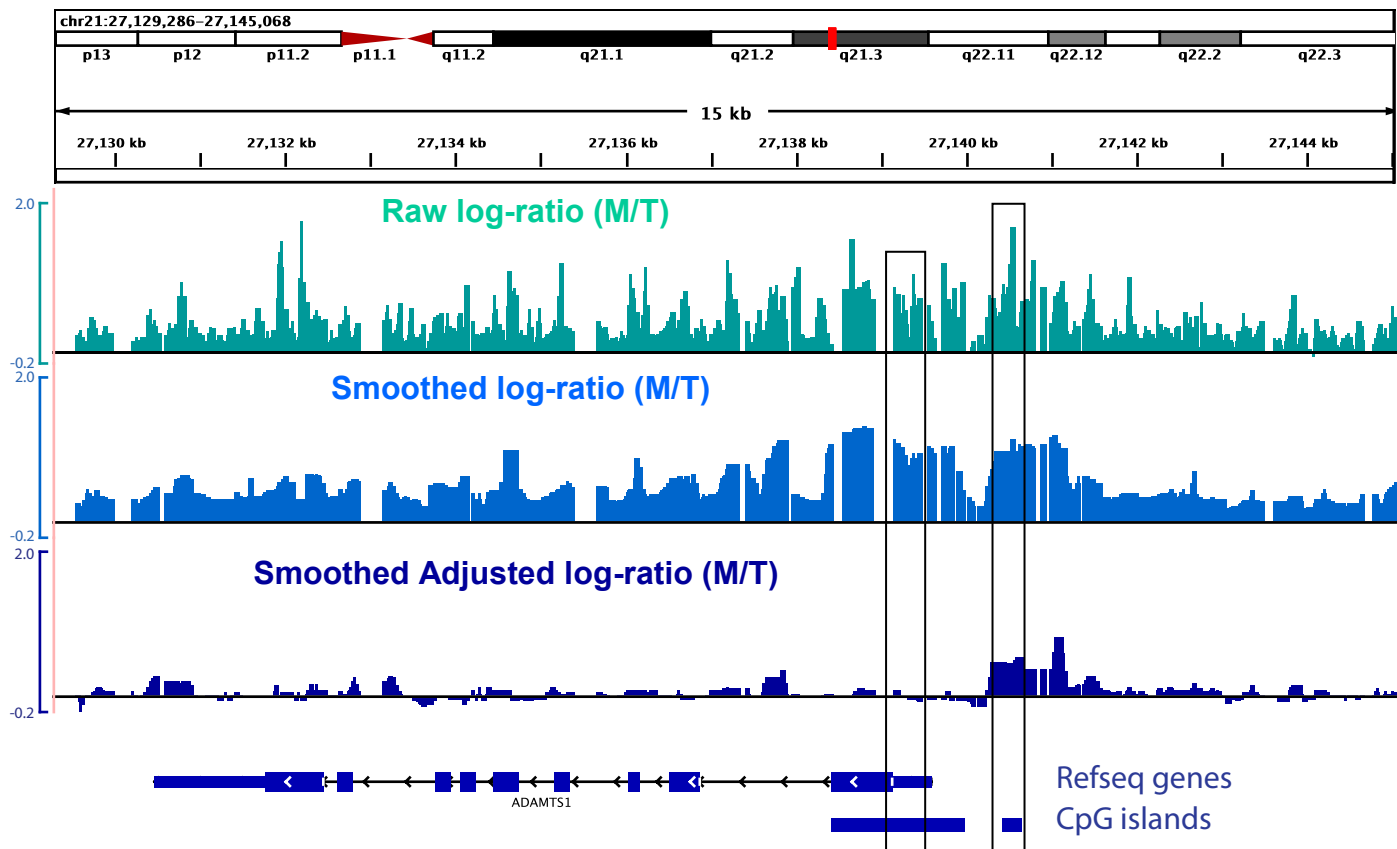

B

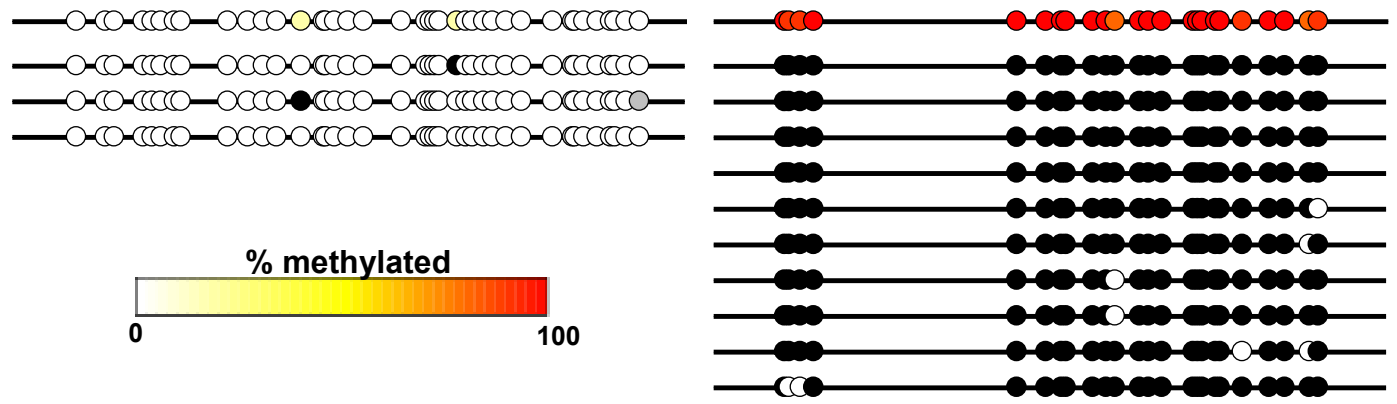

Supplement: Additional file 2 — Representative results and validation of the MBD-chip pre-processing approach. A, The raw log2(M/T), smoothed log2(M/T), and the smoothed adjusted log2(M/T) are shown for LNCaP cells for a representative 15 kbp region on chr 22. The raw and smoothed log2(M/T) appear to be high throughout the region. A running median of the adjusted log2(M/T) attenuates the signal in most regions (e.g., boxed region on the left) but maintains a high signal in a region upstream of the ADAMTS1 gene (boxed region on the right). The shown region is annotated with the chromosome coordinates (top), Refseq genes, and CpG islands. B, Representative results of bisulfite sequencing experiments verifying the accuracy of the smoothed adjusted log2(M/T) as a measure of DNA methylation. Note that the boxed region on the left, which has very low log2(M/T) signals from the microarrays (panel (A)), shows near absence of methylation of the underlying CpG island, while the boxed region on the right, which shows a relatively high log2(M/T) signal from the microarrays (panel (A)), shows nearly complete methylation of the underlying CpG island. Circles represent positions of CpGs. In the top lines for each region, the color of each circle represents the fraction of sequenced alleles that were methylated at that CpG according to the color scale. Each subsequent line represents the methylation pattern for each sequenced clone; black and white circles indicate methylated and unmethylated CpGs respectively. This convention is used for all subsequent bisulfite sequencing figures. [file 1471-2164-12-313-S2.PDF]

MBD-Chip regions called as methylated in LNCaP or PrEC

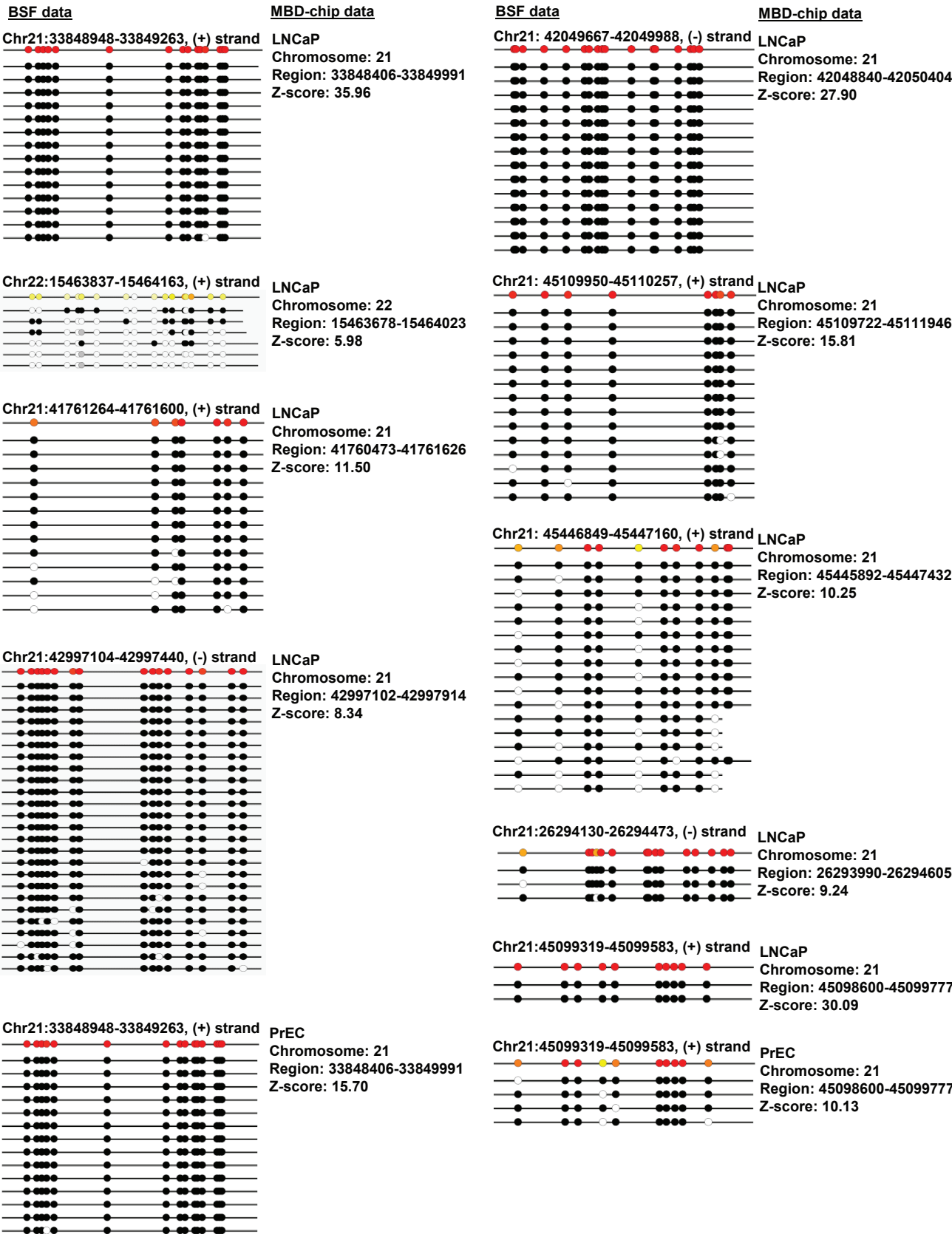

Supplement: Additional file 3 — Bisulfite sequencing verification data of methylated regions identified by MBD-chip in the LNCaP and PrEC samples. The "BSF data" columns show results from bisulfite sequencing of an amplicon (chromosomal coordinates of each bisulfite sequencing amplicon are shown above each region) within the region called by the MBD-chip analysis. For each methylated region identified by the MBD-chip analysis, the cell line, chromosome coordinates, and additive standardized Z-score for each region are listed in the columns labeled "MBD-chip data". Conventions for bisulfite sequencing are the same as those for Additional File 2 panel B. [file 1471-2164-12-313-S3.PDF]

Additional file 7.

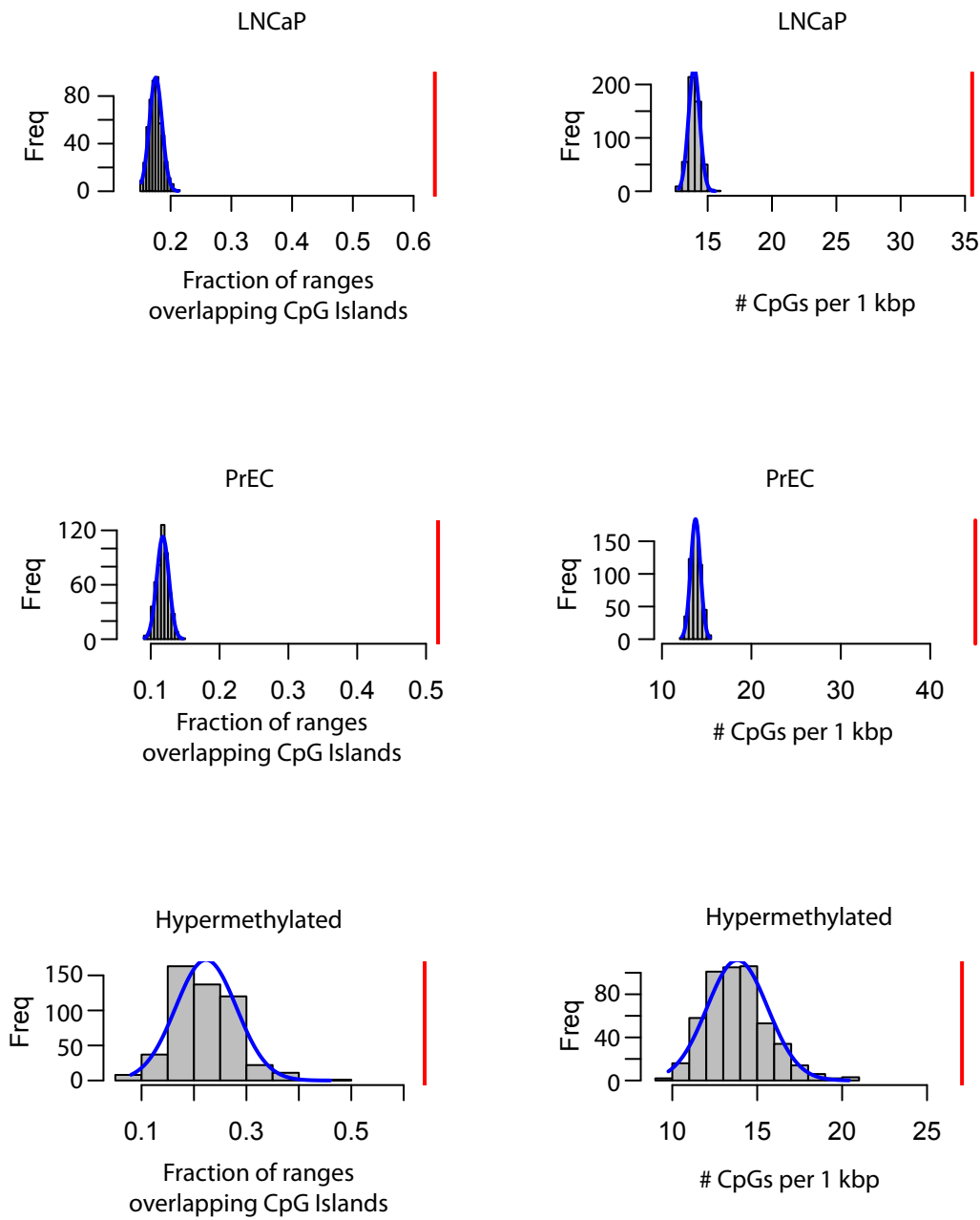

Supplement: Additional file 7 — Identified methylated and hypermethylated regions show a much higher CpG content and overlap with CpG islands than would be expected by random chance. In each panel, the distribution plot shows the expected probability (y-axis) due to random chance of identifying regions with the indicated average fraction of regions overlapping with CpG islands (left panels) or the indicated number of CpGs per 1 kbp (right panels) as plotted on the x-axis. The gray bars represent a non-parametric distribution for the expected probabilities. The overlying blue line represents a best-fit normal distribution of the expected probabilities. The vertical red line indicates actual observed data. [file 1471-2164-12-313-S7.PDF]

# Additional file 8.

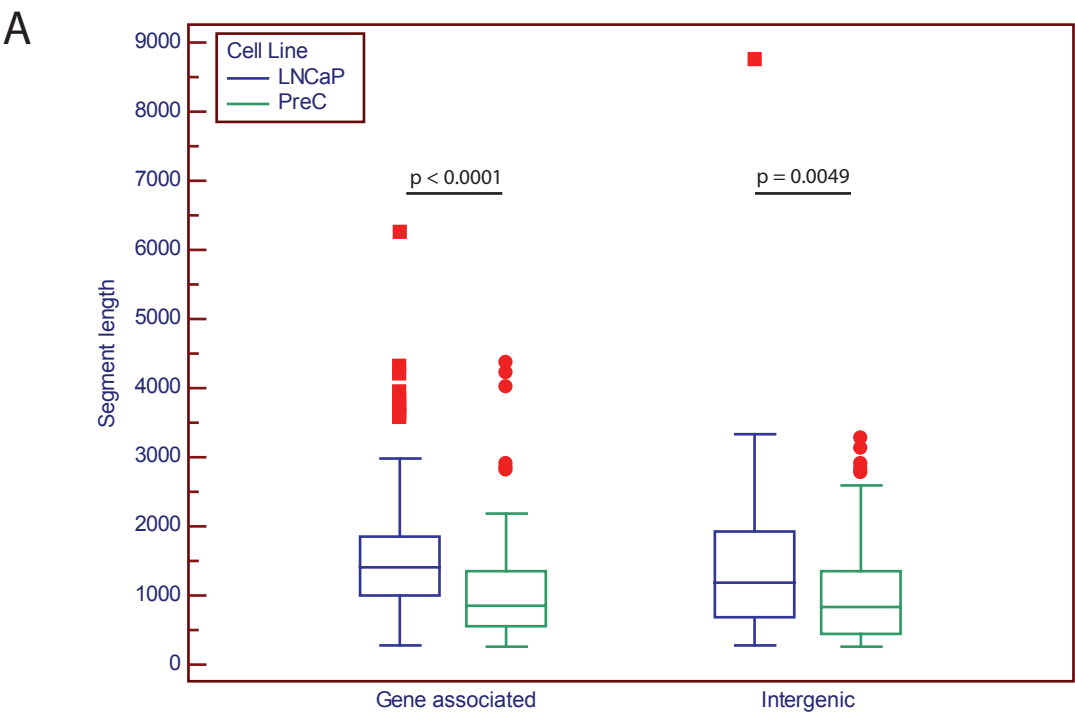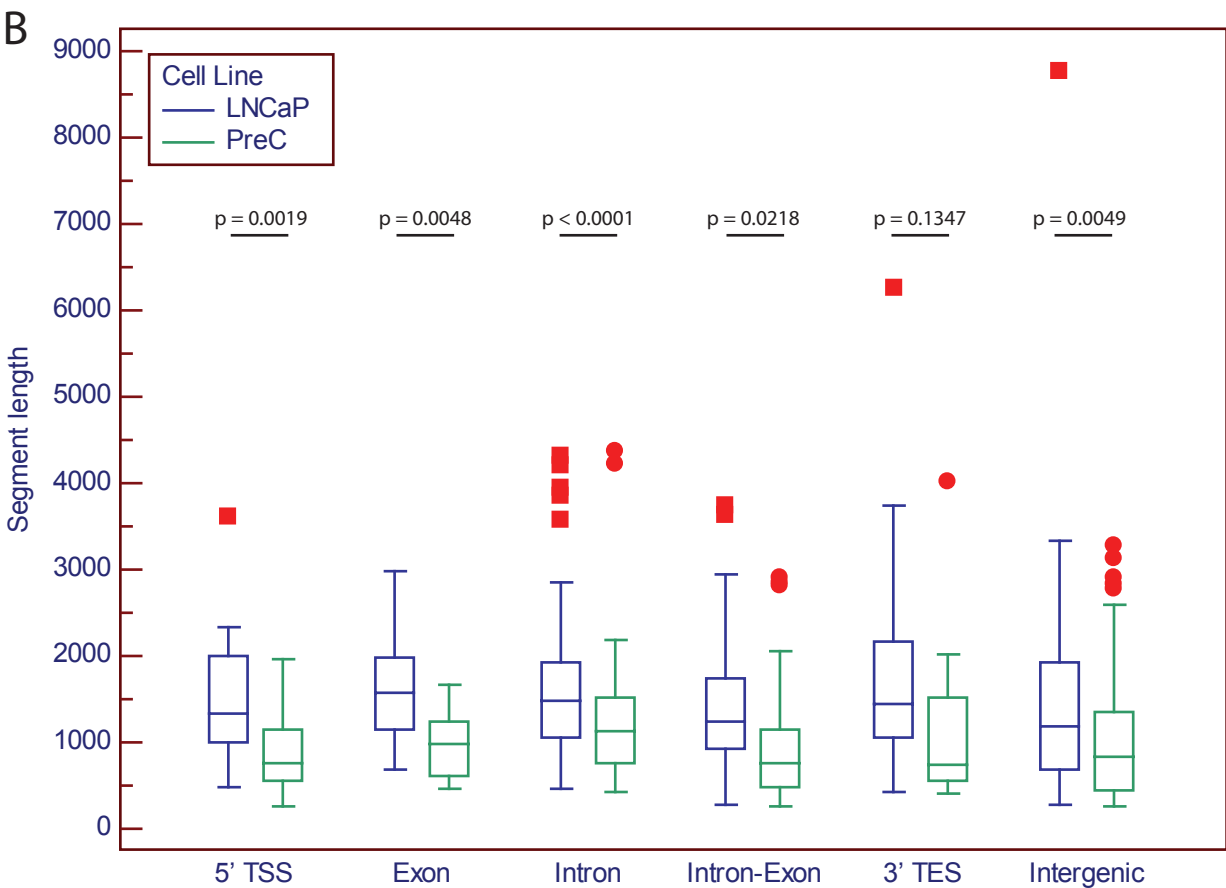

Supplement: Additional file 8 — Segment lengths of methylated regions in LNCaP cells are significantly longer than those of PrEC cells, but do not differ significantly across different genome compartments within each cell line. Shown are box-and-whisker plots representing the distribution of segment lengths of methylated regions. The box represents the 25th to 75th percentile, and the whiskers represent the 5th and 95th percentiles. Red symbols indicate outliers. [file 1471-2164-12-313-S8.PDF]

Additional file 9.

A

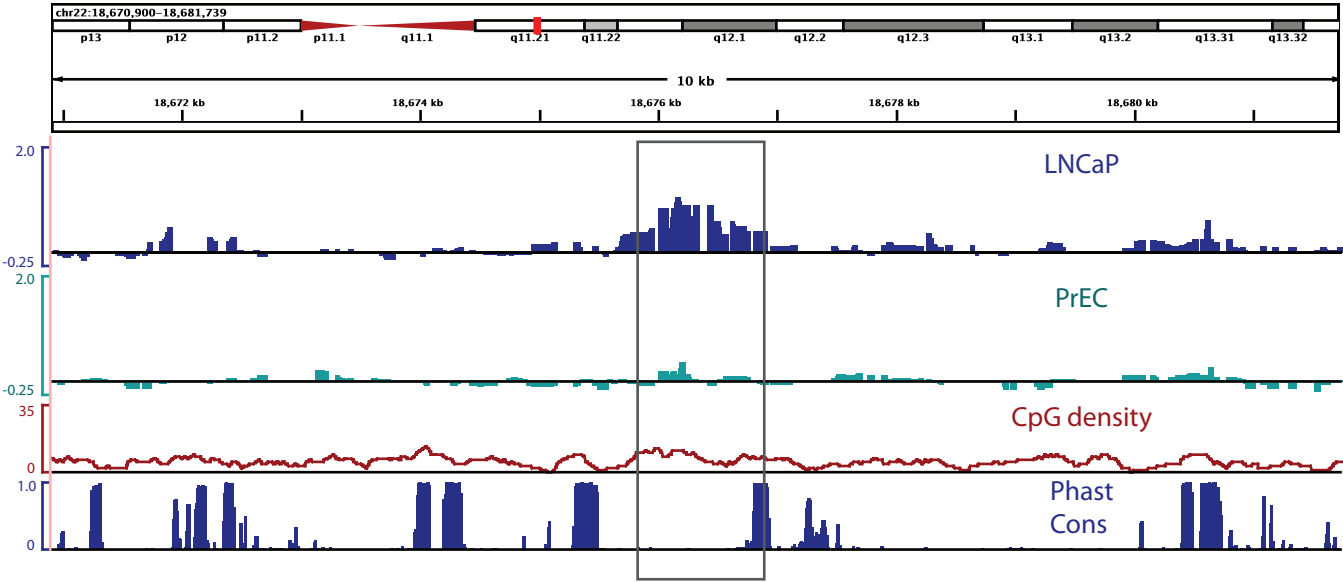

B

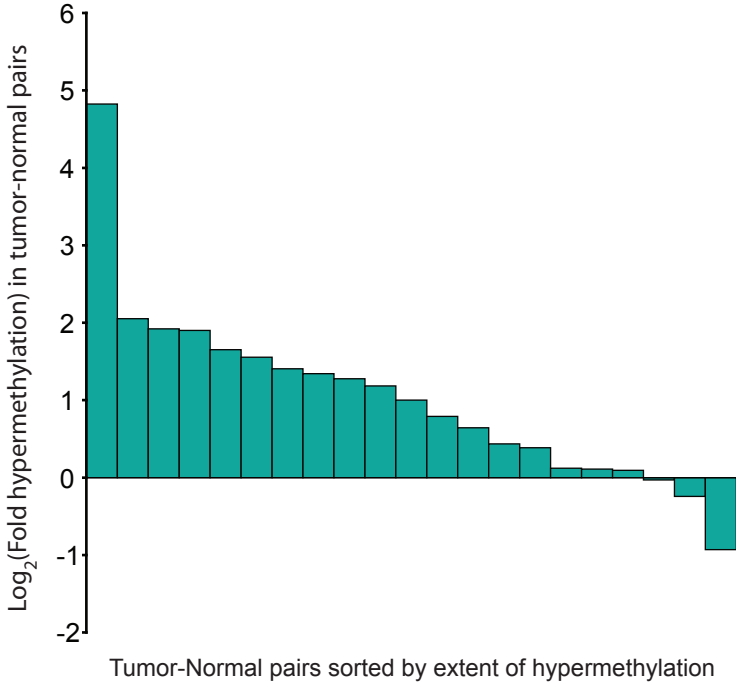

Supplement: Additional file 9 — Frequent hypermethylation of a representative conserved intergenic region. A, DNA methylation signals (smoothed adjusted log2(M/T)) surrounding a representative intergenic region that was identified to be hypermethylated in the LNCaP cells compared to PrEC cells. The shown region is annotated with the chromosome coordinates (top), CpG density (number of CpGs in sliding 250 bp windows), and PhastCons scores. The boxed area represents the region identified to be hypermethylated. Note that the region overlaps sequences with high conservation as indicated by high PhastCons scores. B, A waterfall plot of the extent of hypermethylation of the boxed region from panel (A) in paired tumor-normal prostate tissues. [file 1471-2164-12-313-S9.PDF]
