# Supplementary material for: Chromosome-wide mapping of DNA methylation patterns in normal and malignant prostate cells reveals pervasive methylation of gene-associated and conserved intergenic sequences
Source: BMC Genomics. 2011 Jun 13;12:313. doi: 10.1186/1471-2164-12-313 (PMC3124442; doi:10.1186/1471-2164-12-313)

Additional file 4.

MBD-Chip regions called as hypermethylated in LNCaP vs. PrEC

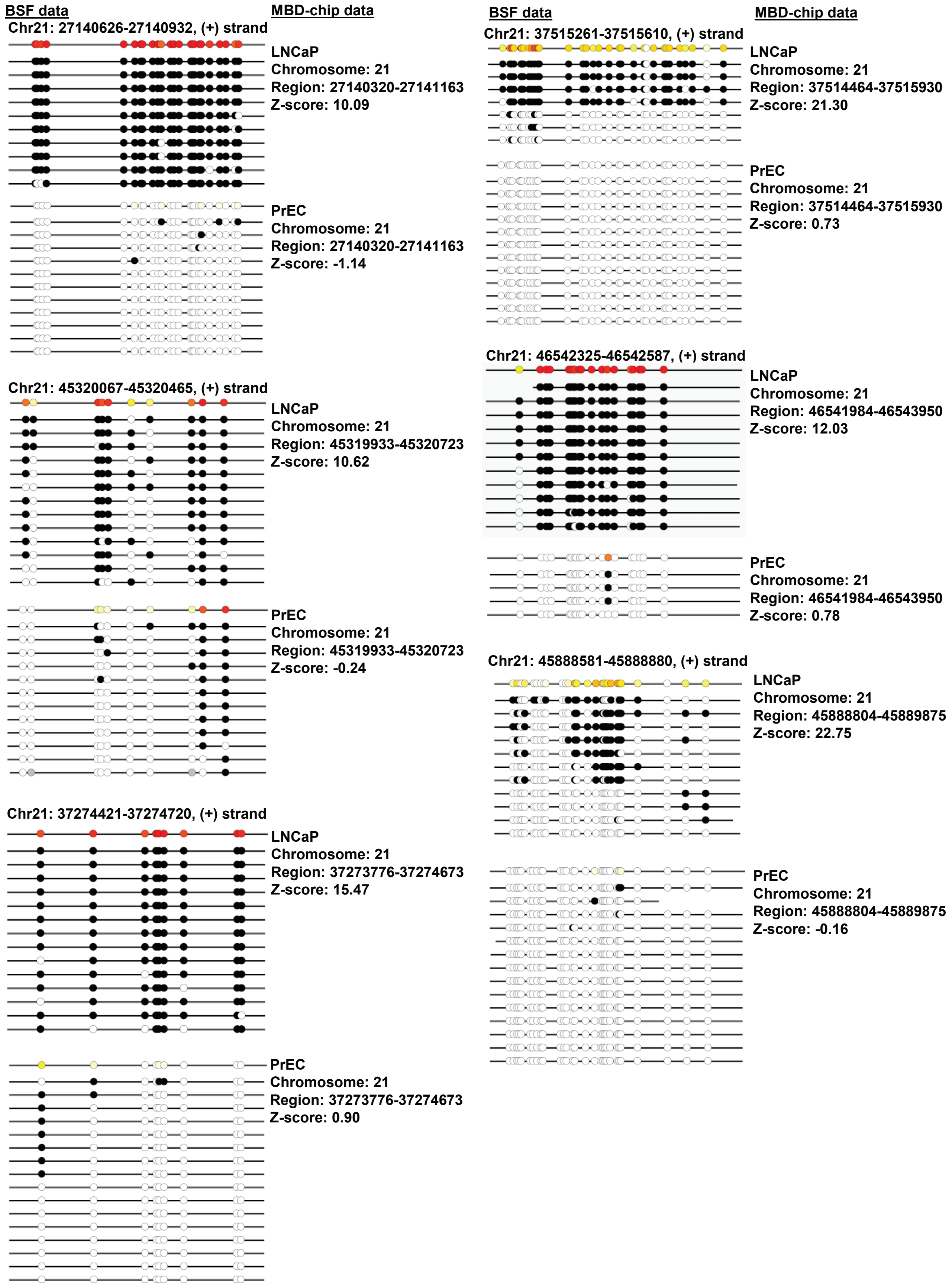

Additional file 4 cont'd.

MBD-Chip regions called as hypermethylated in LNCaP vs. PrEC

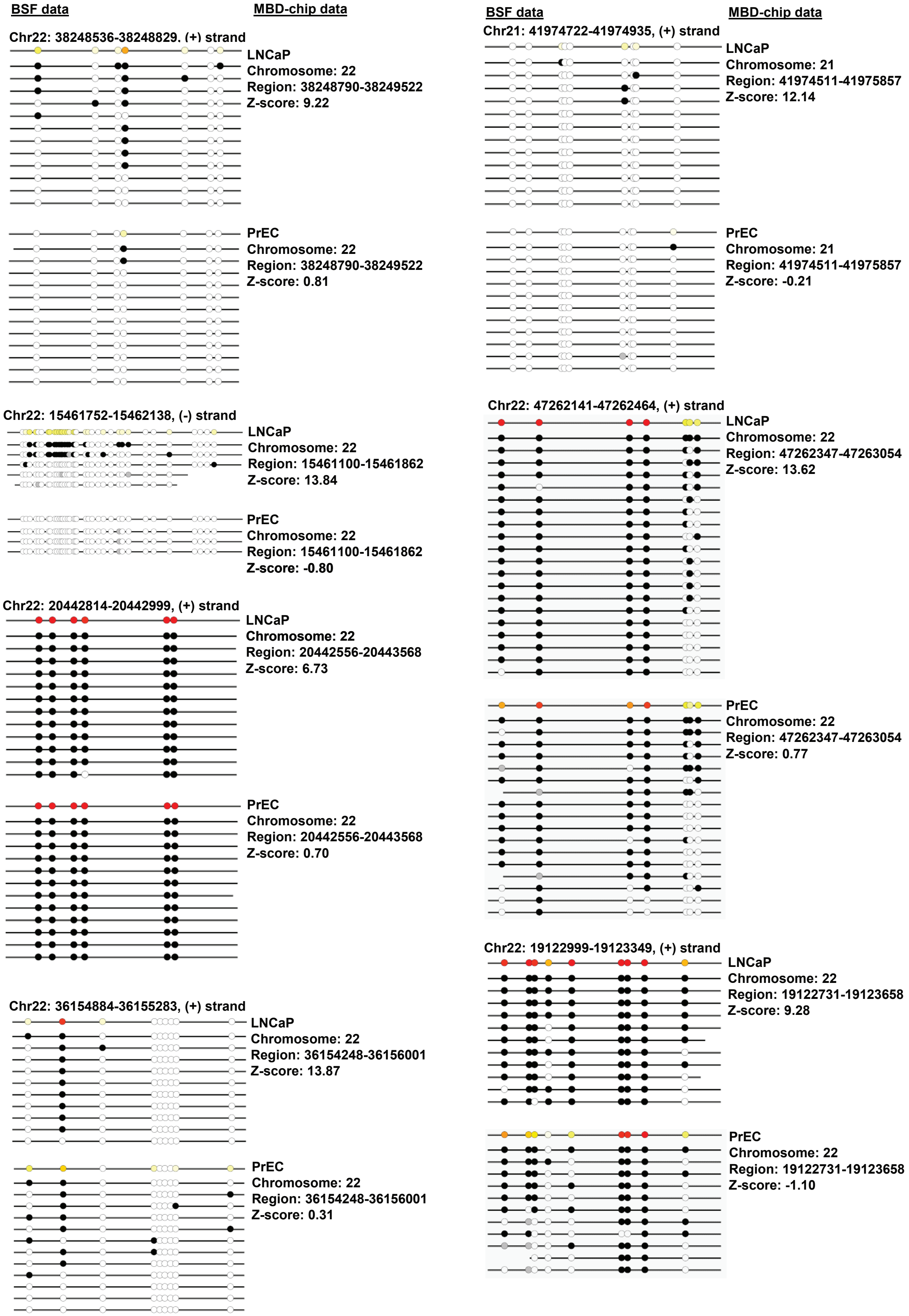

Supplement: Additional file 4 — Bisulfite sequencing verification data of regions that were identified as hypermethylated in the LNCaP compared to the PrEC cells. Conventions are the same as those for Additional File 3. [file 1471-2164-12-313-S4.PDF]
