# Supplementary material for: Chromosome-wide mapping of DNA methylation patterns in normal and malignant prostate cells reveals pervasive methylation of gene-associated and conserved intergenic sequences
Source: BMC Genomics. 2011 Jun 13;12:313. doi: 10.1186/1471-2164-12-313 (PMC3124442; doi:10.1186/1471-2164-12-313)
Supplement: Additional file 5 — Top 150 methylated regions from each of chromosomes 21 and 22 in LNCaP prostate cancer cells and PrEC normal prostate epithelial cells. [file 1471-2164-12-313-S5.PDF]

**Additional file 5. Table A. Top 150 methylated regions from each of chromosomes 21 and 22 in LNCaP prostate cancer cells.**

| Rank | Chromosome[a] | Region Start[a] | Region End[a] | Gene Annotation[b] | Gene Name[c] | Distance to TSS[d] |
|---|---|---|---|---|---|---|
| 1 | chr21 | 46625694 | 46629761 | Intron-Exon | PCNT | 57230 |
| 2 | chr21 | 32593951 | 32595135 | Intron | MRAP | 7956 |
| 3 | chr21 | 46167570 | 46170260 | Intron | PCBP3 | 73267 |
| 4 | chr21 | 42982699 | 42984112 | Intron | PDE9A | 35768 |
| 5 | chr21 | 46646975 | 46648915 | Intron | PCNT | 78511 |
| 6 | chr21 | 33844095 | 33846831 | Exon | SON | 6875 |
| 7 | chr21 | 43141310 | 43143198 | TES | WDR4 | 29549 |
| 8 | chr21 | 33848406 | 33849991 | Intron-Exon | SON | 11186 |
| 9 | chr21 | 44204583 | 44206182 | Intron | AGPAT3 | 95039 |
| 10 | chr21 | 46383879 | 46385374 | Intron | FTCD | 14535 |
| 11 | chr21 | 42930319 | 42931135 | Intergenic | PDE9A | -15796 |
| 12 | chr21 | 44041721 | 44047741 | TES | LOC284837 | 9135 |
| 13 | chr21 | 45098600 | 45099777 | Intron-Exon | PTTG1IP | 18392 |
| 14 | chr21 | 44030600 | 44031730 | TSS | RRP1 | -2116 |
| 15 | chr21 | 36325852 | 36327827 | TES | SETD4 | 26859 |
| 16 | chr21 | 44380809 | 44384411 | Intron-Exon | C21orf33 | 2887 |
| 17 | chr21 | 45589162 | 45590540 | Intergenic | LOC642852 | 56767 |
| 18 | chr21 | 42048840 | 42050404 | Intron-Exon | RIPK4 | 9914 |
| 19 | chr21 | 45218220 | 45219641 | Intron | C21orf70 | 33837 |
| 20 | chr21 | 44161057 | 44161772 | Intron | AGPAT3 | 51513 |
| 21 | chr21 | 45377495 | 45379316 | Intron | ADARB1 | 58574 |
| 22 | chr21 | 13957760 | 13960674 | Intergenic | POTED | 53391 |
| 23 | chr21 | 37813268 | 37813883 | Intergenic | DYRK1A | 98796 |
| 24 | chr21 | 46609357 | 46610617 | Intron | PCNT | 40893 |
| 25 | chr21 | 43485258 | 43488347 | Intergenic | CRYAA | 23048 |
| 26 | chr21 | 43315638 | 43316415 | Intron | PKNOX1 | 47926 |
| 27 | chr21 | 44356789 | 44358599 | Intron-Exon | PWP2 | 5153 |
| 28 | chr21 | 44331018 | 44334970 | Intron-Exon | TRAPPC10 | 74384 |
| 29 | chr21 | 45888804 | 45889875 | Intergenic | SLC19A1 | -102025 |
| 30 | chr21 | 45990206 | 45992868 | Intergenic | PCBP3 | -101435 |
| 31 | chr21 | 16343885 | 16346103 | Intergenic | C21orf34 | -18610 |
| 32 | chr21 | 46674145 | 46676606 | Intron-Exon | PCNT | 105681 |
| 33 | chr21 | 45662676 | 45663740 | TES | NCRNA00175 | 5673 |
| 34 | chr21 | 34188943 | 34190186 | Intergenic | ATP5O | 19842 |
| 35 | chr21 | 37514464 | 37515930 | TES | DSCR9 | 11639 |
| 36 | chr21 | 43203519 | 43203965 | TES | NDUFV3 | 17072 |
| 37 | chr21 | 43999949 | 44001156 | Intron-Exon | PDXK | 36543 |
| 38 | chr21 | 36424415 | 36425251 | Intergenic | CBR3 | -3882 |
| 39 | chr21 | 45551673 | 45552678 | Intergenic | LOC642852 | 19278 |
| 40 | chr21 | 43386203 | 43386903 | Intron-Exon | U2AF1 | 13854 |
| 41 | chr21 | 46663756 | 46666327 | Intron-Exon | PCNT | 95292 |
| 42 | chr21 | 45353707 | 45354249 | Intron | ADARB1 | 34786 |
| 43 | chr21 | 46175985 | 46177637 | Intron | PCBP3 | 81682 |
| 44 | chr21 | 46617699 | 46619582 | Intron | PCNT | 49235 |
| 45 | chr21 | 46424119 | 46424926 | Intron | C21orf56 | 3875 |
| 46 | chr21 | 40674551 | 40675302 | Intron | DSCAM | 465607 |
| 47 | chr21 | 44480319 | 44481261 | Intron-Exon | ICOSLG | 4001 |
| 48 | chr21 | 43349840 | 43351335 | Intron-Exon | CBS | 17774 |
| 49 | chr21 | 39689849 | 39691424 | Intron-Exon | WRB | 15766 |
| 50 | chr21 | 46787400 | 46788260 | Intron | DIP2A | 84110 |
| 51 | chr21 | 46797737 | 46799011 | Intron-Exon | DIP2A | 94447 |
| 52 | chr21 | 44700839 | 44703361 | TES | LRRC3 | 1018 |
| 53 | chr21 | 42848806 | 42849831 | Intron | SLC37A1 | 55995 |
| 54 | chr21 | 43914111 | 43914706 | Intron-Exon | RRP1B | 10251 |
| 55 | chr21 | 46686096 | 46687344 | Intron-Exon | PCNT | 117632 |
| 56 | chr21 | 45464657 | 45465253 | Intron-Exon | ADARB1 | 145736 |
| 57 | chr21 | 46396303 | 46397109 | Intron-Exon | FTCD | 2800 |
| 58 | chr21 | 37273776 | 37274673 | Intron | HLCS | 9733 |
| 59 | chr21 | 46631867 | 46633909 | Intron-Exon | PCNT | 63403 |
| 60 | chr21 | 46597469 | 46598716 | Intron-Exon | PCNT | 29005 |
| 61 | chr21 | 42883572 | 42883937 | Intergenic | PDE9A | -62994 |
| 62 | chr21 | 43199378 | 43199912 | Intron | NDUFV3 | 12931 |
| 63 | chr21 | 44213108 | 44216809 | Intron-Exon | AGPAT3 | 103564 |
| 64 | chr21 | 45718216 | 45719385 | Intron-Exon | COL18A1 | 68691 |
| 65 | chr21 | 37745027 | 37746395 | Intron | DYRK1A | 83298 |
| 66 | chr21 | 25656002 | 25656576 | Intergenic | NCRNA00158 | 69308 |
| 67 | chr21 | 44346801 | 44348439 | Intron-Exon | TRAPPC10 | 90167 |
| 68 | chr21 | 44037990 | 44039669 | Intron-Exon | RRP1 | 4144 |
| 69 | chr21 | 43998338 | 43998966 | Intron | PDXK | 34932 |
| 70 | chr21 | 45419961 | 45420869 | Intron-Exon | ADARB1 | 101040 |
| 71 | chr21 | 44364286 | 44365058 | Intron-Exon | PWP2 | 12650 |
| 72 | chr21 | 39630521 | 39630774 | Intergenic | HMGN1 | 12143 |
| 73 | chr21 | 43353722 | 43355031 | Intron-Exon | CBS | 14078 |
| 74 | chr21 | 44151252 | 44153311 | Intron | AGPAT3 | 41708 |
| 75 | chr21 | 43542541 | 43543913 | Intergenic | CRYAA | 80331 |
| 76 | chr21 | 46669164 | 46670165 | Intron | PCNT | 100700 |
| 77 | chr21 | 37578158 | 37578687 | Intergenic | DSCR3 | -16455 |
| 78 | chr21 | 44095966 | 44096874 | Intergenic | AGPAT3 | -12670 |
| 79 | chr21 | 43809490 | 43809932 | Intron | HSF2BP | 93870 |
| 80 | chr21 | 45093580 | 45094609 | TES | PTTG1IP | 23560 |

| Rank | Chromosome[a] | Region Start[a] | Region End[a] | Gene Annotation[b] | Gene Name[c] | Distance to TSS[d] |
|---|---|---|---|---|---|---|
| 1 | chr22 | 14609123 | 14610311 | Intergenic | POTEH | 57626 |
| 2 | chr22 | 14502350 | 14504397 | Intergenic | POTEH | 163540 |
| 3 | chr22 | 19867148 | 19870053 | Intergenic | POM121L8P | -96661 |
| 4 | chr22 | 22415730 | 22417202 | Exon | ZNF70 | 6077 |
| 5 | chr22 | 19038365 | 19046900 | Intergenic | ZNF74 | -31580 |
| 6 | chr22 | 18273824 | 18275755 | Intron | TXNRD2 | 33604 |
| 7 | chr22 | 17117277 | 17119271 | Intergenic | GGT3P | 40203 |
| 8 | chr22 | 44988670 | 44989989 | Intron-Exon | PPARA | 63507 |
| 9 | chr22 | 14793558 | 14795136 | Intergenic | OR11H1 | 34668 |
| 10 | chr22 | 16107755 | 16108964 | Intergenic | CECR1 | -36976 |
| 11 | chr22 | 18092140 | 18095627 | TES | 5-Sep | 10153 |
| 12 | chr22 | 45773278 | 45773888 | Intron | TBC1D22A | 236065 |
| 13 | chr22 | 30891905 | 30891992 | Intergenic | C22orf42 | -6662 |
| 14 | chr22 | 20106051 | 20109549 | Intron | HIC2 | 4358 |
| 15 | chr22 | 36144508 | 36145958 | Intron | ELFN2 | 7493 |
| 16 | chr22 | 22421924 | 22422099 | TES | VPREB3 | 4531 |
| 17 | chr22 | 45864893 | 45866358 | Intron | TBC1D22A | 327680 |
| 18 | chr22 | 14596271 | 14598497 | Intergenic | POTEH | 69440 |
| 19 | chr22 | 21854160 | 21855219 | Intron-Exon | BCR | 1608 |
| 20 | chr22 | 17498455 | 17499786 | Exon | DGCR14 | -13735 |
| 21 | chr22 | 14635289 | 14636427 | TES | POTEH | 31510 |
| 22 | chr22 | 36449241 | 36451543 | Exon | TRIOBP | 26300 |
| 23 | chr22 | 14784345 | 14785748 | Intergenic | OR11H1 | 44056 |
| 24 | chr22 | 48394920 | 48396142 | Intergenic | C22orf34 | 41052 |
| 25 | chr22 | 20328058 | 20328688 | TES | SDF2L1 | 1516 |
| 26 | chr22 | 45093817 | 45095590 | Intron | GTSE1 | 22515 |
| 27 | chr22 | 14722702 | 14723328 | Intergenic | POTEH | -54765 |
| 28 | chr22 | 48602482 | 48604296 | Intron-Exon | BRD1 | 160 |
| 29 | chr22 | 14475347 | 14475810 | Intergenic | POTEH | 192127 |
| 30 | chr22 | 45690742 | 45692438 | Intron | TBC1D22A | 153529 |
| 31 | chr22 | 14718625 | 14721614 | Intergenic | POTEH | -50688 |
| 32 | chr22 | 45765320 | 45766672 | Intron | TBC1D22A | 228107 |
| 33 | chr22 | 41804515 | 41805427 | Intron | TTLL1 | 9951 |
| 34 | chr22 | 17238487 | 17239507 | Intergenic | DGCR6 | -34229 |
| 35 | chr22 | 21869911 | 21870890 | Intron | BCR | 17359 |
| 36 | chr22 | 14716524 | 14717433 | Intergenic | POTEH | -48587 |
| 37 | chr22 | 21935191 | 21937288 | TES | FBXW4P1 | 237 |
| 38 | chr22 | 19105019 | 19107044 | TES | SCARF2 | 15102 |
| 39 | chr22 | 18120234 | 18121019 | Intergenic | TBX1 | -3207 |
| 40 | chr22 | 22553750 | 22554513 | Intron | SLC2A11 | 24691 |
| 41 | chr22 | 27404866 | 27406652 | TSS | TTC28 | 0 |
| 42 | chr22 | 19810077 | 19812563 | Intergenic | LOC400891 | 79828 |
| 43 | chr22 | 16453965 | 16454926 | TES | ATP6V1E1 | 36662 |
| 44 | chr22 | 23151426 | 23152472 | TSS | ADORA2A | -1058 |
| 45 | chr22 | 17502369 | 17503189 | TES | TSSK2 | 4048 |
| 46 | chr22 | 45148443 | 45150043 | Intron | CELSR1 | 161688 |
| 47 | chr22 | 19448709 | 19449440 | Intron-Exon | PI4KA | -94361 |
| 48 | chr22 | 48663547 | 48664922 | Exon | ZBED4 | 30046 |
| 49 | chr22 | 22959981 | 22960909 | Intron | GGT5 | 10201 |
| 50 | chr22 | 23139738 | 23141316 | Intron-Exon | CYTSA | 142952 |
| 51 | chr22 | 19148093 | 19149096 | Intron | KLHL22 | 31026 |
| 52 | chr22 | 21229032 | 21229983 | TSS | LOC648691 | -1773 |
| 53 | chr22 | 49558627 | 49559138 | Intron | RPL23AP82 | -16758 |
| 54 | chr22 | 22110570 | 22110600 | Intergenic | ZDHHC8P1 | -35771 |
| 55 | chr22 | 17284666 | 17285532 | Intron | PRODH | 18282 |
| 56 | chr22 | 29854529 | 29854740 | Intron-Exon | INPP5J | 5568 |
| 57 | chr22 | 16770596 | 16771487 | Intron | MICAL3 | 115838 |
| 58 | chr22 | 45633448 | 45635480 | Intron | TBC1D22A | 96235 |
| 59 | chr22 | 45006177 | 45006767 | Intron-Exon | PPARA | 81014 |
| 60 | chr22 | 48560938 | 48564292 | TSS | LOC90834 | -976 |
| 61 | chr22 | 19713544 | 19714900 | TES | P2RX6 | 14102 |
| 62 | chr22 | 45401258 | 45403349 | TSS | GRAMD4 | 0 |
| 63 | chr22 | 16256645 | 16256880 | Intergenic | CECR2 | -79748 |
| 64 | chr22 | 18346053 | 18348655 | Intron-Exon | ARVCF | 35654 |
| 65 | chr22 | 15245715 | 15247317 | Intergenic | CCT8L2 | 206383 |
| 66 | chr22 | 39799977 | 39801221 | Intergenic | MIR1281 | -17242 |
| 67 | chr22 | 45096980 | 45097974 | Intron-Exon | GTSE1 | 25678 |
| 68 | chr22 | 15608470 | 15608899 | Intergenic | XKR3 | 73685 |
| 69 | chr22 | 49346861 | 49348039 | Intron | C22orf41 | 155 |
| 70 | chr22 | 15998740 | 15999691 | Intron-Exon | CECR5 | 20478 |
| 71 | chr22 | 45238190 | 45239569 | Intron-Exon | CELSR1 | 72162 |
| 72 | chr22 | 20386473 | 20388531 | Intron-Exon | YPEL1 | 31540 |
| 73 | chr22 | 43975136 | 43976658 | TSS | MIR1249 | 0 |
| 74 | chr22 | 43982536 | 43983504 | Intron | C22orf9 | 3507 |
| 75 | chr22 | 16681704 | 16683052 | Intron-Exon | MICAL3 | 204273 |
| 76 | chr22 | 18247790 | 18248994 | Intron-Exon | TXNRD2 | 60365 |
| 77 | chr22 | 43540788 | 43541721 | Intron | PRR5-ARHGAP8 | 63742 |
| 78 | chr22 | 23350134 | 23351896 | Intron | GGT1 | 40416 |
| 79 | chr22 | 48566897 | 48567790 | Intron-Exon | BRD1 | 36666 |
| 80 | chr22 | 20364496 | 20365776 | Intron-Exon | PPIL2 | 14223 |

| Rank | Chromosome[a] | Region Start[a] | Region End[a] | Gene Annotation[b] | Gene Name[c] | Distance to TSS[d] |
|---|---|---|---|---|---|---|
| 81 | chr21 | 46511379 | 46511862 | Intron-Exon | MCM3AP | 17802 |
| 82 | chr21 | 44677110 | 44677816 | Intron | TRPM2 | 79198 |
| 83 | chr21 | 32706381 | 32707947 | TSS | C21orf63 | 0 |
| 84 | chr21 | 39549237 | 39549696 | Intron-Exon | BRWD1 | 57730 |
| 85 | chr21 | 44164217 | 44164484 | Intron | AGPAT3 | 54673 |
| 86 | chr21 | 45232564 | 45234928 | Intergenic | NCRNA00162 | 14142 |
| 87 | chr21 | 46892516 | 46894053 | Intron-Exon | PRMT2 | 12561 |
| 88 | chr21 | 44009741 | 44011979 | Intergenic | CSTB | 8708 |
| 89 | chr21 | 45390225 | 45390648 | Intron | ADARB1 | 71304 |
| 90 | chr21 | 15324857 | 15326456 | Intron | NRIP1 | 32541 |
| 91 | chr21 | 13902573 | 13903062 | TSS | POTED | -1307 |
| 92 | chr21 | 13903466 | 13904803 | TSS | POTED | 0 |
| 93 | chr21 | 43977879 | 43978622 | Intron-Exon | PDXK | 14473 |
| 94 | chr21 | 45144869 | 45147364 | Intron-Exon | ITGB2 | 18029 |
| 95 | chr21 | 43937860 | 43938819 | Exon | RRP1B | 34000 |
| 96 | chr21 | 46451142 | 46452731 | Intron-Exon | LSS | 20435 |
| 97 | chr21 | 46939783 | 46939813 | Intergenic | PRMT2 | 59828 |
| 98 | chr21 | 42127665 | 42129766 | Intron-Exon | PRDM15 | 42885 |
| 99 | chr21 | 45109722 | 45111946 | Intron-Exon | PTTG1IP | 6223 |
| 100 | chr21 | 45511082 | 45512262 | Intron-Exon | POFUT2 | 19977 |
| 101 | chr21 | 43724026 | 43725765 | TSS | C21orf84 | -1495 |
| 102 | chr21 | 43153313 | 43154555 | Intron | WDR4 | 18192 |
| 103 | chr21 | 44228159 | 44228695 | Exon | AGPAT3 | 118615 |
| 104 | chr21 | 45204456 | 45205210 | Intron-Exon | C21orf70 | 20073 |
| 105 | chr21 | 45055585 | 45055614 | Intron | SUMO3 | 6858 |
| 106 | chr21 | 16397894 | 16398222 | Intron | C21orf34 | 33181 |
| 107 | chr21 | 43632853 | 43633789 | Intergenic | SIK1 | 37641 |
| 108 | chr21 | 44111375 | 44112038 | Intron | AGPAT3 | 1831 |
| 109 | chr21 | 44858088 | 44859468 | TES | KRTAP10-8 | 1664 |
| 110 | chr21 | 43155424 | 43156797 | Intron-Exon | WDR4 | 15950 |
| 111 | chr21 | 44372500 | 44374248 | Intron-Exon | PWP2 | 20864 |
| 112 | chr21 | 46448846 | 46450730 | Intron-Exon | LSS | 22436 |
| 113 | chr21 | 44412114 | 44412986 | Intergenic | C21orf33 | 34192 |
| 114 | chr21 | 45097474 | 45098306 | Intron | PTTG1IP | 19863 |
| 115 | chr21 | 43994614 | 43996363 | Intron-Exon | PDXK | 31208 |
| 116 | chr21 | 16340772 | 16341265 | Intergenic | C21orf34 | -23448 |
| 117 | chr21 | 43996626 | 43998067 | Intron-Exon | PDXK | 33220 |
| 118 | chr21 | 44316499 | 44317374 | Intron | TRAPPC10 | 59865 |
| 119 | chr21 | 36589282 | 36590345 | TES | DOPEY2 | 130573 |
| 120 | chr21 | 43063824 | 43064644 | Intron-Exon | PDE9A | 116893 |
| 121 | chr21 | 42552427 | 42553914 | Intron | ABCG1 | 59559 |
| 122 | chr21 | 37227462 | 37228349 | Intron | HLCS | 56057 |
| 123 | chr21 | 10162747 | 10162768 | Intergenic | BAGE | -41939 |
| 124 | chr21 | 44229533 | 44230552 | Exon | AGPAT3 | 119989 |
| 125 | chr21 | 46454972 | 46457054 | Intron-Exon | LSS | 16112 |
| 126 | chr21 | 44575767 | 44577238 | Intron-Exon | C21orf2 | 6475 |
| 127 | chr21 | 36288115 | 36288657 | Intergenic | SETD4 | 65824 |
| 128 | chr21 | 42978194 | 42980058 | Intron | PDE9A | 31263 |
| 129 | chr21 | 42985852 | 42987185 | Intron | PDE9A | 38921 |
| 130 | chr21 | 36524806 | 36525242 | Exon | DOPEY2 | 66097 |
| 131 | chr21 | 44211795 | 44212387 | Intron-Exon | AGPAT3 | 102251 |
| 132 | chr21 | 43048674 | 43050164 | Intron | PDE9A | 101743 |
| 133 | chr21 | 44177938 | 44179546 | Intron | AGPAT3 | 68394 |
| 134 | chr21 | 44053380 | 44055054 | Exon | LOC284837 | 1822 |
| 135 | chr21 | 42663810 | 42665054 | TES | TMPRSS3 | 24215 |
| 136 | chr21 | 41752427 | 41753159 | TES | MX1 | 38037 |
| 137 | chr21 | 44184908 | 44186202 | Intron | AGPAT3 | 75364 |
| 138 | chr21 | 33564344 | 33565479 | Intron | IL10RB | 3802 |
| 139 | chr21 | 46435715 | 46436516 | Intron-Exon | LSS | 36650 |
| 140 | chr21 | 43466666 | 43467857 | TES | CRYAA | 4456 |
| 141 | chr21 | 45467648 | 45468422 | Intron | ADARB1 | 148727 |
| 142 | chr21 | 36990737 | 36991238 | TSS | SIM2 | -2623 |
| 143 | chr21 | 42383032 | 42383585 | Intron-Exon | UMODL1 | -19090 |
| 144 | chr21 | 45683728 | 45685664 | Intron | COL18A1 | 34203 |
| 145 | chr21 | 14365548 | 14365684 | Intergenic | C21orf81 | -90912 |
| 146 | chr21 | 43764525 | 43764643 | Intergenic | C21orf84 | -41994 |
| 147 | chr21 | 45715224 | 45716899 | Intron | COL18A1 | 65699 |
| 148 | chr21 | 42039992 | 42040678 | Intron-Exon | RIPK4 | 19640 |
| 149 | chr21 | 14304246 | 14305142 | Intergenic | C21orf81 | -29610 |
| 150 | chr21 | 44232544 | 44233956 | TES | AGPAT3 | 123000 |

| Rank | Chromosome[a] | Region Start[a] | Region End[a] | Gene Annotation[b] | Gene Name[c] | Distance to TSS[d] |
|---|---|---|---|---|---|---|
| 81 | chr22 | 32419850 | 32419882 | Intron | LARGE | 226534 |
| 82 | chr22 | 48869920 | 48870699 | TSS | MOV10L1 | 0 |
| 83 | chr22 | 43538873 | 43540499 | Intron | PRR5-ARHGAP8 | 61827 |
| 84 | chr22 | 41772304 | 41772924 | Intron-Exon | TTLL1 | 42454 |
| 85 | chr22 | 45155579 | 45156604 | Intron-Exon | CELSR1 | 155127 |
| 86 | chr22 | 19267731 | 19269084 | Intron-Exon | MED15 | 75845 |
| 87 | chr22 | 36805697 | 36806548 | Intron | SLC16A8 | 2568 |
| 88 | chr22 | 45933189 | 45933219 | Intron | TBC1D22A | 395976 |
| 89 | chr22 | 45165279 | 45166035 | Intron-Exon | CELSR1 | 145696 |
| 90 | chr22 | 36154248 | 36156001 | TSS | ELFN2 | -797 |
| 91 | chr22 | 16665961 | 16666965 | Intron | MICAL3 | 220360 |
| 92 | chr22 | 49495687 | 49497213 | Intron-Exon | SHANK3 | 35751 |
| 93 | chr22 | 45144196 | 45145145 | Intron-Exon | CELSR1 | 166586 |
| 94 | chr22 | 18087638 | 18088654 | TSS | GP1BB | -2412 |
| 95 | chr22 | 16230278 | 16231086 | Intergenic | CECR2 | -105542 |
| 96 | chr22 | 15461100 | 15461862 | TSS | psiTPTE22 | -939 |
| 97 | chr22 | 47262347 | 47263054 | TSS | FAM19A5 | -898 |
| 98 | chr22 | 35017696 | 35021024 | Intron-Exon | MYH9 | 92985 |
| 99 | chr22 | 38856794 | 38857324 | Intron | TNRC6B | 86027 |
| 100 | chr22 | 18341427 | 18343141 | Intron-Exon | ARVCF | 41168 |
| 101 | chr22 | 48640728 | 48641891 | Intron | ZBED4 | 7227 |
| 102 | chr22 | 18471992 | 18472469 | Intron | DGCR8 | 24158 |
| 103 | chr22 | 48398312 | 48398807 | TES | C22orf34 | 38387 |
| 104 | chr22 | 42874690 | 42875388 | Intron-Exon | PARVB | 148184 |
| 105 | chr22 | 45126464 | 45127465 | Intron-Exon | TRMU | 16502 |
| 106 | chr22 | 42742013 | 42742043 | Intron | PARVB | 15507 |
| 107 | chr22 | 18675688 | 18676880 | Intergenic | DGCR6L | 10728 |
| 108 | chr22 | 48250490 | 48250734 | Intergenic | C22orf34 | 186460 |
| 109 | chr22 | 36951281 | 36953234 | Intron-Exon | TMEM184B | 45728 |
| 110 | chr22 | 48946040 | 48946285 | Intergenic | PANX2 | -5002 |
| 111 | chr22 | 21795264 | 21796174 | Intron | RTDR1 | -18977 |
| 112 | chr22 | 41359951 | 41359980 | Intron | CYB5R3 | 10604 |
| 113 | chr22 | 39903812 | 39904786 | Exon | EP300 | 85252 |
| 114 | chr22 | 37182699 | 37182928 | TSS | KCNJ4 | -1550 |
| 115 | chr22 | 22387327 | 22388252 | Intron-Exon | LOC91316 | 1358 |
| 116 | chr22 | 22612100 | 22613641 | Intergenic | GSTT2B | 19727 |
| 117 | chr22 | 18592240 | 18592303 | Intergenic | LOC150197 | 18385 |
| 118 | chr22 | 46555296 | 46557085 | Intergenic | FLJ46257 | -149314 |
| 119 | chr22 | 45154495 | 45155162 | Intron | CELSR1 | 156569 |
| 120 | chr22 | 16790519 | 16791478 | Intron | MICAL3 | 95847 |
| 121 | chr22 | 30997435 | 30999135 | Intergenic | SLC5A4 | -16117 |
| 122 | chr22 | 49013905 | 49014464 | Intron-Exon | TUBGCP6 | 11063 |
| 123 | chr22 | 18506862 | 18507318 | Intron-Exon | ZDHHC8 | 7497 |
| 124 | chr22 | 45681015 | 45682276 | Intron | TBC1D22A | 143802 |
| 125 | chr22 | 23876571 | 23876762 | Intron | KIAA1671 | 122630 |
| 126 | chr22 | 24083401 | 24083802 | Intron | LRP5L | 4722 |
| 127 | chr22 | 34065913 | 34066343 | Intron | TOM1 | 40645 |
| 128 | chr22 | 49009132 | 49010348 | Intron-Exon | TUBGCP6 | 15179 |
| 129 | chr22 | 49464783 | 49468160 | Intron | SHANK3 | 4847 |
| 130 | chr22 | 17423441 | 17424446 | Intron | DGCR2 | 65521 |
| 131 | chr22 | 17409364 | 17410463 | Intron-Exon | DGCR2 | 79504 |
| 132 | chr22 | 38754594 | 38755767 | Intron-Exon | FAM83F | 33695 |
| 133 | chr22 | 44001023 | 44002200 | Intron | C22orf9 | 13114 |
| 134 | chr22 | 14608439 | 14608868 | Intergenic | POTEH | 59069 |
| 135 | chr22 | 18684370 | 18685067 | Intron | DGCR6L | 2541 |
| 136 | chr22 | 29281435 | 29282156 | Intron-Exon | GAL3ST1 | 8720 |
| 137 | chr22 | 18453945 | 18454133 | TES | MIR1306 | 364 |
| 138 | chr22 | 16651186 | 16653079 | Exon | MICAL3 | 234246 |
| 139 | chr22 | 49251814 | 49252767 | Intron-Exon | SBF1 | 7563 |
| 140 | chr22 | 39936843 | 39937107 | Intron | L3MBTL2 | 5584 |
| 141 | chr22 | 23383133 | 23385157 | TSS | POM121L10P | 0 |
| 142 | chr22 | 41376883 | 41377268 | TSS | CYB5R3 | -1534 |
| 143 | chr22 | 36858254 | 36858662 | Intron | PLA2G6 | 49045 |
| 144 | chr22 | 41898267 | 41898944 | Intron-Exon | TTLL12 | 14107 |
| 145 | chr22 | 22973844 | 22974930 | TSS | POM121L9P | -2659 |
| 146 | chr22 | 43970535 | 43971237 | Exon | C22orf9 | 15774 |
| 147 | chr22 | 33208386 | 33208417 | Intergenic | LARGE | -561970 |
| 148 | chr22 | 26531236 | 26531584 | Intergenic | MN1 | -3750 |
| 149 | chr22 | 14535182 | 14537929 | Intergenic | POTEH | 130008 |
| 150 | chr22 | 43706894 | 43708116 | Intron | PHF21B | 76129 |

a, Chromosomal coordinates are derived from the UCSC hg18 genome build

b, TSS, overlaps a 3 kbp region upstream of the transcriptional start site of a gene; TES, overlaps a 3 kbp region dowstream of the transcriptional termination site of a gene; Exon, region is entirely contained within a gene exon; Intron, region is entirely contained within a gene intron; Intron-Exon, region overlaps an intron-exon boundary

c, Gene associated with the annotation shown in the Gene annotation column. In the case of regions annotated as intergenic, the nearest gene is shown.

d, Distance (in bp) to the transcriptional start site of the gene shown in the Gene name column. If region falls upstream of the TSS, then this will be a negative integer; if region falls downstream of the TSS, then this will be a positive integer; if region overlaps the TSS, then the value is zero.

## Additional file 5. Table B. Top 150 methylated regions from each of chromosomes 21 and 22 in PrEC normal prostate epithelial cells

| Rank | Chromosome[a] | Region Start[a] | Region End[a] | Gene Annotation[b] | Gene Name[c] | Distance to TSS[d] |
|---|---|---|---|---|---|---|
| 1 | chr21 | 46625759 | 46629729 | Intron-Exon | PCNT | 57295 |
| 2 | chr21 | 32594015 | 32595105 | Intron | MRAP | 8020 |
| 3 | chr21 | 36325852 | 36327317 | TES | SETD4 | 27369 |
| 4 | chr21 | 45663245 | 45663740 | TES | NCRNA00175 | 5673 |
| 5 | chr21 | 19538750 | 19540196 | Intergenic | PRSS7 | -840909 |
| 6 | chr21 | 13957713 | 13960596 | Intergenic | POTED | 53344 |
| 7 | chr21 | 46303662 | 46304644 | Intergenic | COL6A2 | -37817 |
| 8 | chr21 | 42930349 | 42930971 | Intergenic | PDE9A | -15960 |
| 9 | chr21 | 46175923 | 46177637 | Intron | PCBP3 | 81620 |
| 10 | chr21 | 46647108 | 46648888 | Intron | PCNT | 78644 |
| 11 | chr21 | 45377952 | 45379316 | Intron | ADARB1 | 59031 |
| 12 | chr21 | 46167661 | 46170219 | Intron | PCBP3 | 73358 |
| 13 | chr21 | 46609357 | 46610617 | Intron | PCNT | 40893 |
| 14 | chr21 | 25656002 | 25656576 | Intergenic | NCRNA00158 | 69308 |
| 15 | chr21 | 44030626 | 44031730 | TSS | RRP1 | -2116 |
| 16 | chr21 | 10128545 | 10128994 | Intergenic | BAGE | -7737 |
| 17 | chr21 | 42982816 | 42983643 | Intron | PDE9A | 35885 |
| 18 | chr21 | 44044483 | 44046413 | Intron-Exon | RRP1 | 10637 |
| 19 | chr21 | 32041603 | 32041628 | Intergenic | SFRS15 | -15301 |
| 20 | chr21 | 43141373 | 43143137 | TES | WDR4 | 29610 |
| 21 | chr21 | 13990679 | 13992331 | Intergenic | POTED | 86310 |
| 22 | chr21 | 43485258 | 43487174 | Intergenic | CRYAA | 23048 |
| 23 | chr21 | 44095933 | 44096977 | Intergenic | AGPAT3 | -12567 |
| 24 | chr21 | 45589328 | 45590540 | Intergenic | LOC642852 | 56933 |
| 25 | chr21 | 43203584 | 43203931 | TES | NDUFV3 | 17137 |
| 26 | chr21 | 13903487 | 13904803 | TSS | POTED | 0 |
| 27 | chr21 | 43350587 | 43351335 | Intron-Exon | CBS | 17774 |
| 28 | chr21 | 14057581 | 14059104 | Intergenic | C21orf15 | 83452 |
| 29 | chr21 | 13902573 | 13903062 | TSS | POTED | -1307 |
| 30 | chr21 | 46396529 | 46396999 | Intron | FTCD | 2910 |
| 31 | chr21 | 44502447 | 44503938 | Intron-Exon | DNMT3L | 2589 |
| 32 | chr21 | 45990175 | 45992827 | Intergenic | PCBP3 | -101476 |
| 33 | chr21 | 33848618 | 33849580 | Intron-Exon | SON | 11398 |
| 34 | chr21 | 10162747 | 10162768 | Intergenic | BAGE | -41939 |
| 35 | chr21 | 45688190 | 45688967 | Intron | COL18A1 | 38665 |
| 36 | chr21 | 45718248 | 45719385 | Intron-Exon | COL18A1 | 68723 |
| 37 | chr21 | 46383967 | 46385374 | Intron | FTCD | 14535 |
| 38 | chr21 | 33845661 | 33846769 | Exon | SON | 8441 |
| 39 | chr21 | 44204583 | 44206043 | Intron | AGPAT3 | 95039 |
| 40 | chr21 | 46892746 | 46894023 | Intron-Exon | PRMT2 | 12791 |
| 41 | chr21 | 32880701 | 32881254 | TSS | TCP10L | -985 |
| 42 | chr21 | 14357976 | 14358473 | Intergenic | C21orf81 | -83340 |
| 43 | chr21 | 43999949 | 44001092 | Intron-Exon | PDXK | 36543 |
| 44 | chr21 | 14303000 | 14305142 | Intergenic | C21orf81 | -28364 |
| 45 | chr21 | 34189058 | 34190186 | Intergenic | ATP5O | 19842 |
| 46 | chr21 | 43542747 | 43543841 | Intergenic | CRYAA | 80537 |
| 47 | chr21 | 46597809 | 46598804 | Intron-Exon | PCNT | 29345 |
| 48 | chr21 | 45742968 | 45743340 | Intron | COL18A1 | 93443 |
| 49 | chr21 | 44412174 | 44412747 | Intergenic | C21orf33 | 34252 |
| 50 | chr21 | 10191640 | 10191703 | Intergenic | BAGE | -70832 |
| 51 | chr21 | 44356789 | 44358542 | Intron-Exon | PWP2 | 5153 |
| 52 | chr21 | 14372747 | 14373324 | Intergenic | C21orf81 | -98111 |
| 53 | chr21 | 44203796 | 44204243 | Intron-Exon | AGPAT3 | 94252 |
| 54 | chr21 | 44994226 | 44995261 | Intergenic | C21orf29 | -38303 |
| 55 | chr21 | 46797799 | 46798567 | Intron-Exon | DIP2A | 94509 |
| 56 | chr21 | 14121029 | 14122089 | Intergenic | C21orf15 | 20467 |
| 57 | chr21 | 46630220 | 46630748 | Intron-Exon | PCNT | 61756 |
| 58 | chr21 | 43636939 | 43637855 | Intergenic | SIK1 | 33575 |
| 59 | chr21 | 46111400 | 46112702 | Intron | PCBP3 | 17097 |
| 60 | chr21 | 45551878 | 45552573 | Intergenic | LOC642852 | 19483 |
| 61 | chr21 | 46312745 | 46312856 | Intergenic | COL6A2 | -29605 |
| 62 | chr21 | 44555437 | 44556128 | Intron | PFKL | 11084 |
| 63 | chr21 | 44381609 | 44382750 | Intron | C21orf33 | 3687 |
| 64 | chr21 | 43632959 | 43633789 | Intergenic | SIK1 | 37641 |
| 65 | chr21 | 44373199 | 44373885 | Intron | PWP2 | 21563 |
| 66 | chr21 | 40674891 | 40675139 | Intron | DSCAM | 465770 |
| 67 | chr21 | 15859100 | 15859128 | Intergenic | USP25 | -165239 |
| 68 | chr21 | 42598206 | 42599309 | Intergenic | TFF3 | 9466 |
| 69 | chr21 | 14368705 | 14369419 | Intergenic | C21orf81 | -94069 |
| 70 | chr21 | 33619021 | 33619376 | TSS | IFNAR1 | 0 |
| 71 | chr21 | 10191672 | 10191825 | Intergenic | BAGE | -70864 |
| 72 | chr21 | 46632237 | 46633676 | Intron-Exon | PCNT | 63773 |
| 73 | chr21 | 10119582 | 10119919 | Intron | BAGE4 | 877 |
| 74 | chr21 | 44771337 | 44771714 | Intron-Exon | C21orf29 | 184209 |
| 75 | chr21 | 41022521 | 41022521 | Intron | DSCAM | 118388 |
| 76 | chr21 | 45467793 | 45468296 | Intron | ADARB1 | 148872 |
| 77 | chr21 | 43937893 | 43938617 | Exon | RRP1B | 34033 |
| 78 | chr21 | 43998282 | 43998966 | Intron | PDXK | 34876 |
| 79 | chr21 | 46669393 | 46669990 | Intron | PCNT | 100929 |
| 80 | chr21 | 23083522 | 23083557 | Intergenic | NCAM2 | 1791018 |

| Rank | Chromosome[a] | Region Start[a] | Region End[a] | Gene Annotation[b] | Gene Name[c] | Distance to TSS[d] |
|---|---|---|---|---|---|---|
| 1 | chr22 | 18091945 | 18095711 | TES | SEPT5 | 9958 |
| 2 | chr22 | 18274222 | 18275788 | Intron | TXNRD2 | 33571 |
| 3 | chr22 | 45985437 | 45988022 | Intergenic | FLJ46257 | 417960 |
| 4 | chr22 | 14609209 | 14610168 | Intergenic | POTEH | 57769 |
| 5 | chr22 | 44988724 | 44989859 | Intron-Exon | PPARA | 63561 |
| 6 | chr22 | 14502933 | 14503710 | Intergenic | POTEH | 164227 |
| 7 | chr22 | 48800541 | 48803197 | Intron | TTLL8 | 31985 |
| 8 | chr22 | 45844051 | 45845541 | Intron | TBC1D22A | 306838 |
| 9 | chr22 | 31055501 | 31057350 | Intergenic | RFPL3 | -23522 |
| 10 | chr22 | 47697915 | 47699011 | Intergenic | FAM19A5 | 347133 |
| 11 | chr22 | 19038626 | 19040972 | Intergenic | ZNF74 | -37508 |
| 12 | chr22 | 42305602 | 42307205 | Intron | EFCAB6 | 232246 |
| 13 | chr22 | 16108432 | 16108964 | Intergenic | CECR1 | -37653 |
| 14 | chr22 | 17117277 | 17119421 | Intergenic | GGT3P | 40053 |
| 15 | chr22 | 22415838 | 22417080 | Exon | ZNF70 | 6199 |
| 16 | chr22 | 19867531 | 19870053 | Intergenic | POM121L8P | -96661 |
| 17 | chr22 | 36448167 | 36452282 | Intron-Exon | TRIOBP | 25226 |
| 18 | chr22 | 19043846 | 19046875 | Intergenic | ZNF74 | -31605 |
| 19 | chr22 | 48394920 | 48396003 | Intergenic | C22orf34 | 41191 |
| 20 | chr22 | 45773278 | 45773769 | Intron | TBC1D22A | 236065 |
| 21 | chr22 | 14635323 | 14636427 | TES | POTEH | 31510 |
| 22 | chr22 | 17239277 | 17239507 | Intergenic | DGCR6 | -34229 |
| 23 | chr22 | 38034613 | 38034645 | Intergenic | SNORD83B | 5217 |
| 24 | chr22 | 19810077 | 19810808 | Intergenic | LOC400891 | 79828 |
| 25 | chr22 | 14596637 | 14598342 | Intergenic | POTEH | 69595 |
| 26 | chr22 | 41804538 | 41805457 | Intron | TTLL1 | 9921 |
| 27 | chr22 | 15608470 | 15608899 | Intergenic | XKR3 | 73685 |
| 28 | chr22 | 20888022 | 20888525 | Intergenic | VPREB1 | -40675 |
| 29 | chr22 | 39800060 | 39800422 | Intergenic | MIR1281 | -18041 |
| 30 | chr22 | 47900656 | 47900977 | Intergenic | C22orf34 | 536217 |
| 31 | chr22 | 48602870 | 48603694 | Exon | BRD1 | 762 |
| 32 | chr22 | 17498552 | 17499966 | Exon | DGCR14 | -13638 |
| 33 | chr22 | 48411005 | 48411792 | Intron | C22orf34 | 25402 |
| 34 | chr22 | 21318611 | 21318638 | TSS | GGTLC2 | -144 |
| 35 | chr22 | 27404941 | 27406652 | TSS | TTC28 | 0 |
| 36 | chr22 | 45094198 | 45095632 | Intron | GTSE1 | 22896 |
| 37 | chr22 | 15247139 | 15247317 | Intergenic | CCT8L2 | 206383 |
| 38 | chr22 | 48820180 | 48820526 | Intron | TTLL8 | 14656 |
| 39 | chr22 | 29281617 | 29281953 | Exon | GAL3ST1 | 8923 |
| 40 | chr22 | 14475470 | 14475810 | Intergenic | POTEH | 192127 |
| 41 | chr22 | 45633530 | 45635341 | Intron | TBC1D22A | 96317 |
| 42 | chr22 | 19811095 | 19812563 | Intergenic | LOC400891 | 80846 |
| 43 | chr22 | 47419859 | 47420432 | Intron | FAM19A5 | 155907 |
| 44 | chr22 | 15380950 | 15382267 | Intergenic | CCT8L2 | 71433 |
| 45 | chr22 | 36100281 | 36101162 | Exon | ELFN2 | 52289 |
| 46 | chr22 | 49464783 | 49467383 | Intron | SHANK3 | 4847 |
| 47 | chr22 | 18608236 | 18608413 | TES | RTN4R | 27403 |
| 48 | chr22 | 41376907 | 41377237 | TSS | CYB5R3 | -1558 |
| 49 | chr22 | 21869911 | 21870890 | Intron | BCR | 17359 |
| 50 | chr22 | 20979006 | 20979801 | TSS | LOC96610 | -2662 |
| 51 | chr22 | 21934782 | 21936141 | TSS | FBXW4P1 | 0 |
| 52 | chr22 | 45125119 | 45125927 | Intron | TRMU | 15157 |
| 53 | chr22 | 45747351 | 45748054 | Intron | TBC1D22A | 210138 |
| 54 | chr22 | 15964377 | 15965370 | Intron-Exon | IL17RA | 18528 |
| 55 | chr22 | 16663677 | 16663884 | Intron | MICAL3 | 223441 |
| 56 | chr22 | 43701019 | 43701526 | Intron | PHF21B | 82719 |
| 57 | chr22 | 48567161 | 48567454 | Intron | BRD1 | 37002 |
| 58 | chr22 | 47574788 | 47575573 | Intergenic | FAM19A5 | 224006 |
| 59 | chr22 | 45765320 | 45766287 | Intron | TBC1D22A | 228107 |
| 60 | chr22 | 33600395 | 33600395 | Intergenic | ISX | -191735 |
| 61 | chr22 | 30146638 | 30146669 | Intron | DRG1 | 21099 |
| 62 | chr22 | 45238190 | 45238982 | Intron-Exon | CELSR1 | 72749 |
| 63 | chr22 | 14784466 | 14785264 | Intergenic | OR11H1 | 44540 |
| 64 | chr22 | 19374624 | 19375830 | Exon | POM121L4P | 781 |
| 65 | chr22 | 19106414 | 19106901 | TES | SCARF2 | 15245 |
| 66 | chr22 | 48945201 | 48946342 | TES | MOV10L1 | 74639 |
| 67 | chr22 | 45411193 | 45411787 | Intron | GRAMD4 | 9871 |
| 68 | chr22 | 48987980 | 48988654 | Intron | SELO | 6445 |
| 69 | chr22 | 40540590 | 40540627 | Intron | CCDC134 | 13966 |
| 70 | chr22 | 45792606 | 45793179 | Intron | TBC1D22A | 255393 |
| 71 | chr22 | 26530463 | 26530727 | TSS | MN1 | -2977 |
| 72 | chr22 | 49216307 | 49218098 | Intron-Exon | SAPS2 | 87681 |
| 73 | chr22 | 15252429 | 15253263 | Intergenic | CCT8L2 | 200437 |
| 74 | chr22 | 19713680 | 19713963 | TES | P2RX6 | 14238 |
| 75 | chr22 | 45144882 | 45145088 | Intron | CELSR1 | 166643 |
| 76 | chr22 | 45898364 | 45898534 | Intron | TBC1D22A | 361151 |
| 77 | chr22 | 43540430 | 43540944 | Intron | PRR5-ARHGAP8 | 63384 |
| 78 | chr22 | 49491500 | 49491704 | Intron | SHANK3 | 31564 |
| 79 | chr22 | 23151204 | 23152441 | TSS | ADORA2A | -1089 |
| 80 | chr22 | 48663631 | 48664267 | Exon | ZBED4 | 30130 |

| Rank | Chromosome[a] | Region Start[a] | Region End[a] | Gene Annotation[b] | Gene Name[c] | Distance to TSS[d] |
|---|---|---|---|---|---|---|
| 81 | chr21 | 44331778 | 44332964 | Intron-Exon | TRAPPC10 | 75144 |
| 82 | chr21 | 29286768 | 29286801 | Intron | RNF160 | 347 |
| 83 | chr21 | 45118507 | 45118507 | TSS | PTTG1IP | -338 |
| 84 | chr21 | 45098693 | 45099634 | Intron-Exon | PTTG1IP | 18535 |
| 85 | chr21 | 46674177 | 46675646 | Intron-Exon | PCNT | 105713 |
| 86 | chr21 | 32240023 | 32241603 | Intron-Exon | HUNK | 72524 |
| 87 | chr21 | 27056190 | 27056218 | Intergenic | ADAMTS1 | 83381 |
| 88 | chr21 | 45197081 | 45198829 | Intron | C21orf70 | 12698 |
| 89 | chr21 | 45078149 | 45078685 | Intergenic | SUMO3 | -15677 |
| 90 | chr21 | 41473559 | 41473586 | Intron | BACE2 | -11988 |
| 91 | chr21 | 46244149 | 46244375 | Intron | COL6A1 | 18058 |
| 92 | chr21 | 44347632 | 44348376 | Exon | TRAPPC10 | 90998 |
| 93 | chr21 | 44858088 | 44859408 | TES | KRTAP10-8 | 1664 |
| 94 | chr21 | 45218177 | 45218760 | Intron | C21orf70 | 33794 |
| 95 | chr21 | 42880388 | 42880621 | Intergenic | PDE9A | -66310 |
| 96 | chr21 | 25963101 | 25963133 | Intron | JAM2 | 29641 |
| 97 | chr21 | 42955541 | 42956732 | Intron | PDE9A | 8610 |
| 98 | chr21 | 44677110 | 44677622 | Intron | TRPM2 | 79198 |
| 99 | chr21 | 45626199 | 45626725 | Intergenic | COL18A1 | -22800 |
| 100 | chr21 | 41788088 | 41788273 | Intron-Exon | TMPRSS2 | 13589 |
| 101 | chr21 | 45674211 | 45674848 | Intron | COL18A1 | 24686 |
| 102 | chr21 | 44537456 | 44538727 | Intron-Exon | AIRE | 7265 |
| 103 | chr21 | 45192522 | 45192895 | Intron | C21orf70 | 8139 |
| 104 | chr21 | 44333796 | 44334133 | Intron | TRAPPC10 | 77162 |
| 105 | chr21 | 14364773 | 14365684 | Intergenic | C21orf81 | -90137 |
| 106 | chr21 | 46108053 | 46108543 | Intron | PCBP3 | 13750 |
| 107 | chr21 | 37578158 | 37578687 | Intergenic | DSCR3 | -16455 |
| 108 | chr21 | 46686278 | 46687076 | Intron-Exon | PCNT | 117814 |
| 109 | chr21 | 44364387 | 44364689 | Intron-Exon | PWP2 | 12751 |
| 110 | chr21 | 45570959 | 45572222 | Intergenic | LOC642852 | 38564 |
| 111 | chr21 | 46238518 | 46239371 | Intron-Exon | COL6A1 | 12427 |
| 112 | chr21 | 46140743 | 46141891 | Intron | PCBP3 | 46440 |
| 113 | chr21 | 45723336 | 45724226 | Intron | COL18A1 | 73811 |
| 114 | chr21 | 45288225 | 45288856 | Intergenic | C21orf122 | 28698 |
| 115 | chr21 | 32731492 | 32731492 | Intron | C21orf63 | 24869 |
| 116 | chr21 | 45223616 | 45224097 | TES | C21orf70 | 39233 |
| 117 | chr21 | 44709634 | 44710690 | Intergenic | LRRC3 | 9813 |
| 118 | chr21 | 46389099 | 46389416 | Intron | FTCD | 10493 |
| 119 | chr21 | 32357639 | 32357663 | Intergenic | NCRNA00159 | -16837 |
| 120 | chr21 | 45776239 | 45776450 | Exon | SLC19A1 | 10329 |
| 121 | chr21 | 41598483 | 41599134 | Intergenic | FAM3B | -11397 |
| 122 | chr21 | 44360283 | 44361211 | Intron | PWP2 | 8647 |
| 123 | chr21 | 46676106 | 46676606 | Intron-Exon | PCNT | 107642 |
| 124 | chr21 | 44185907 | 44186172 | Intron | AGPAT3 | 76363 |
| 125 | chr21 | 44041813 | 44042943 | Intron-Exon | RRP1 | 7967 |
| 126 | chr21 | 45232970 | 45233329 | Intergenic | NCRNA00162 | 15741 |
| 127 | chr21 | 45146482 | 45147364 | Intron | ITGB2 | 18029 |
| 128 | chr21 | 37367492 | 37368133 | TSS | PIGP | -164 |
| 129 | chr21 | 14017464 | 14018628 | Intergenic | POTED | 113095 |
| 130 | chr21 | 45684721 | 45685311 | Intron | COL18A1 | 35196 |
| 131 | chr21 | 44228431 | 44228695 | Exon | AGPAT3 | 118887 |
| 132 | chr21 | 22690369 | 22690400 | Intergenic | NCAM2 | 1397865 |
| 133 | chr21 | 14305397 | 14305918 | Intergenic | C21orf81 | -30761 |
| 134 | chr21 | 44152077 | 44152438 | Intron | AGPAT3 | 42533 |
| 135 | chr21 | 45420203 | 45420757 | Exon | ADARB1 | 101282 |
| 136 | chr21 | 45743658 | 45744062 | Intron | COL18A1 | 94133 |
| 137 | chr21 | 32292535 | 32292984 | Intron-Exon | HUNK | 125036 |
| 138 | chr21 | 44795712 | 44796228 | TSS | KRTAP10-2 | 0 |
| 139 | chr21 | 45781016 | 45781299 | Intron | SLC19A1 | 5480 |
| 140 | chr21 | 14370808 | 14371076 | Intergenic | C21orf81 | -96172 |
| 141 | chr21 | 44489903 | 44490357 | TES | DNMT3L | 16170 |
| 142 | chr21 | 46182903 | 46182992 | Intron | PCBP3 | 88600 |
| 143 | chr21 | 42883572 | 42883727 | Intergenic | PDE9A | -63204 |
| 144 | chr21 | 41752455 | 41752841 | Exon | MX1 | 38065 |
| 145 | chr21 | 35086514 | 35087291 | Intron-Exon | RUNX1 | 95566 |
| 146 | chr21 | 43386242 | 43386763 | Intron-Exon | U2AF1 | 13994 |
| 147 | chr21 | 46787616 | 46788194 | Intron | DIP2A | 84326 |
| 148 | chr21 | 33021626 | 33022473 | TSS | SYNJ1 | 0 |
| 149 | chr21 | 42579891 | 42580203 | Intron | ABCG1 | 87023 |
| 150 | chr21 | 13973631 | 13973817 | Intergenic | POTED | 69262 |

| Rank | Chromosome[a] | Region Start[a] | Region End[a] | Gene Annotation[b] | Gene Name[c] | Distance to TSS[d] |
|---|---|---|---|---|---|---|
| 81 | chr22 | 34278435 | 34279256 | Exon | RASD2 | 11137 |
| 82 | chr22 | 45391786 | 45391878 | Intergenic | GRAMD4 | -9444 |
| 83 | chr22 | 47526254 | 47526499 | Intron | FAM19A5 | 262302 |
| 84 | chr22 | 49346833 | 49348067 | Intron | C22orf41 | 127 |
| 85 | chr22 | 45126609 | 45127001 | Intron-Exon | TRMU | 16647 |
| 86 | chr22 | 45402648 | 45403464 | Intron | GRAMD4 | 1326 |
| 87 | chr22 | 45758031 | 45758031 | Intron | TBC1D22A | 220818 |
| 88 | chr22 | 28992175 | 28992553 | Intron | OSM | 287 |
| 89 | chr22 | 33782433 | 33782455 | Intergenic | ISX | -9675 |
| 90 | chr22 | 48398534 | 48398807 | TES | C22orf34 | 38387 |
| 91 | chr22 | 39937078 | 39937107 | Intron | L3MBTL2 | 5819 |
| 92 | chr22 | 43707095 | 43707983 | Intron | PHF21B | 76262 |
| 93 | chr22 | 45006207 | 45006681 | Intron-Exon | PPARA | 81044 |
| 94 | chr22 | 45627066 | 45627598 | Intron | TBC1D22A | 89853 |
| 95 | chr22 | 43681874 | 43682270 | Intron | PHF21B | 101975 |
| 96 | chr22 | 26289502 | 26289534 | Intergenic | MN1 | 237952 |
| 97 | chr22 | 18085975 | 18086728 | Intron | 40426 | 3988 |
| 98 | chr22 | 34065913 | 34066238 | Intron | TOM1 | 40645 |
| 99 | chr22 | 18506565 | 18507285 | Intron-Exon | ZDHHC8 | 7200 |
| 100 | chr22 | 37153873 | 37154097 | Exon | KCNJ4 | 15882 |
| 101 | chr22 | 26400367 | 26400367 | Intergenic | MN1 | 127119 |
| 102 | chr22 | 48640858 | 48641891 | Intron | ZBED4 | 7357 |
| 103 | chr22 | 45931294 | 45931745 | Intron | TBC1D22A | 394081 |
| 104 | chr22 | 46658486 | 46658689 | Intergenic | FLJ46257 | -252504 |
| 105 | chr22 | 35836937 | 35837101 | Intergenic | TMPRSS6 | -7298 |
| 106 | chr22 | 17502966 | 17503123 | TES | TSSK2 | 4645 |
| 107 | chr22 | 45936604 | 45937196 | Intron | TBC1D22A | 399391 |
| 108 | chr22 | 30891905 | 30891992 | Intergenic | C22orf42 | -6662 |
| 109 | chr22 | 47524386 | 47524671 | Intron | FAM19A5 | 260434 |
| 110 | chr22 | 20246006 | 20247096 | Intergenic | UBE2L3 | -4861 |
| 111 | chr22 | 21901997 | 21902101 | Intron | BCR | 49445 |
| 112 | chr22 | 22347812 | 22347910 | Intron | LOC91316 | -41798 |
| 113 | chr22 | 25289610 | 25289610 | Intron | TPST2 | 1760 |
| 114 | chr22 | 45400421 | 45401313 | TSS | GRAMD4 | -9 |
| 115 | chr22 | 45284258 | 45284258 | Intron | CELSR1 | 27473 |
| 116 | chr22 | 48808339 | 48809074 | Intron | TTLL8 | 26108 |
| 117 | chr22 | 21229057 | 21229357 | TSS | LOC648691 | -2399 |
| 118 | chr22 | 18087739 | 18088254 | TSS | GP1BB | -2812 |
| 119 | chr22 | 42035458 | 42036197 | Intron | SCUBE1 | 33102 |
| 120 | chr22 | 39800690 | 39801221 | Intergenic | MIR1281 | -17242 |
| 121 | chr22 | 25159868 | 25160642 | Intron-Exon | ASPHD2 | 4588 |
| 122 | chr22 | 43341472 | 43341607 | TSS | NCRNA00207 | -2277 |
| 123 | chr22 | 45155691 | 45156309 | Intron | CELSR1 | 155422 |
| 124 | chr22 | 48469991 | 48470344 | Intergenic | C22orf34 | -32797 |
| 125 | chr22 | 47461048 | 47461079 | Intron | FAM19A5 | 197096 |
| 126 | chr22 | 49496572 | 49497299 | Intron-Exon | SHANK3 | 36636 |
| 127 | chr22 | 49460749 | 49461127 | Intron | SHANK3 | 813 |
| 128 | chr22 | 36845909 | 36845939 | Intron | PLA2G6 | 61768 |
| 129 | chr22 | 15944588 | 15944959 | TSS | IL17RA | -890 |
| 130 | chr22 | 17315463 | 17315500 | Intergenic | PRODH | -11649 |
| 131 | chr22 | 47409212 | 47409241 | Intron | FAM19A5 | 145260 |
| 132 | chr22 | 42356150 | 42356180 | Intron | EFCAB6 | 183271 |
| 133 | chr22 | 41772328 | 41772593 | Intron-Exon | TTLL1 | 42785 |
| 134 | chr22 | 17873784 | 17873885 | Intron | CDC45 | 26368 |
| 135 | chr22 | 22975401 | 22975588 | TSS | POM121L9P | -2001 |
| 136 | chr22 | 36704118 | 36704161 | Intron | SOX10 | 6324 |
| 137 | chr22 | 17353450 | 17354503 | Intron | DGCR5 | 15423 |
| 138 | chr22 | 15387653 | 15388107 | Intergenic | CCT8L2 | 65593 |
| 139 | chr22 | 30430910 | 30430936 | Intron | C22orf30 | 45184 |
| 140 | chr22 | 48857344 | 48857694 | Intron-Exon | MLC1 | 8214 |
| 141 | chr22 | 49222494 | 49223388 | Intron-Exon | SAPS2 | 93868 |
| 142 | chr22 | 47418753 | 47419005 | Intron | FAM19A5 | 154801 |
| 143 | chr22 | 16445344 | 16445627 | Intron-Exon | SLC25A18 | 22161 |
| 144 | chr22 | 44102928 | 44102992 | Intron | FAM118A | 19183 |
| 145 | chr22 | 38285318 | 38285318 | Intergenic | CACNA1I | -11386 |
| 146 | chr22 | 29457936 | 29457967 | Intron | OSBP2 | 37143 |
| 147 | chr22 | 45165279 | 45165603 | Intron | CELSR1 | 146128 |
| 148 | chr22 | 46405603 | 46406499 | TSS | FLJ46257 | 0 |
| 149 | chr22 | 45194130 | 45195225 | Intron | CELSR1 | 116506 |
| 150 | chr22 | 20364496 | 20365776 | Intron-Exon | PPIL2 | 14223 |

a, Chromosomal coordinates are derived from the UCSC hg18 genome build

b, TSS, overlaps a 3 kbp region upstream of the transcriptional start site of a gene; TES, overlaps a 3 kbp region dowstream of the transcriptional termination site of a gene; Exon, region is entirely contained within a gene exon; Intron, region is entirely contained within a gene intron; Intron-Exon, region overlaps an intron-exon boundary

c, Gene associated with the annotation shown in the Gene annotation column. In the case of regions annotated as intergenic, the nearest gene is shown.

d, Distance (in bp) to the transcriptional start site of the gene shown in the Gene name column. If region falls upstream of the TSS, then this will be a negative integer; if region falls downstream of the TSS, then this will be a positive integer; if region overlaps the TSS, then the value is zero.
