# Supplementary material for: Chromosome-wide mapping of DNA methylation patterns in normal and malignant prostate cells reveals pervasive methylation of gene-associated and conserved intergenic sequences
Source: BMC Genomics. 2011 Jun 13;12:313. doi: 10.1186/1471-2164-12-313 (PMC3124442; doi:10.1186/1471-2164-12-313)
Supplement: Additional file 6 — Top 25 regions from each of chromosomes 21 and 22 that are hypermethylated in LNCaP vs. PrEC. [file 1471-2164-12-313-S6.PDF]

Additional file 6.

Top 25 regions from each of chromosomes 21 and 22 hypermethylated in LNCaP vs. PrEC

Rank	Chromosome [a]	Region Start [a]	Region End [a]	Gene Annotation [b]	Gene Name [c]	Distance to nearest TSS [d]
1	chr21	45888804	45889875	Intergenic	SLC19A1	-102025
2	chr21	37514464	37515930	TES	DSCR9	11639
3	chr21	45109722	45111946	Intron-Exon	PTTG1IP	6223
4	chr21	37273776	37274673	Intron	HLCS	9733
5	chr21	44053380	44055054	Exon	LOC284837	1822
6	chr21	42042441	42043060	Intron	RIPK4	17258
7	chr21	44111375	44112038	Intron	AGPAT3	1831
8	chr21	46291752	46292971	Intergenic	COL6A2	-49490
9	chr21	43977879	43978622	Intron-Exon	PDXK	14473
10	chr21	41974511	41975857	Intron	NCRNA00111	1980
11	chr21	41760473	41761626	Intron-Exon	TMPRSS2	40236
12	chr21	44671278	44672466	Intron-Exon	TRPM2	73366
13	chr21	46541984	46543950	TES	C21orf57	11289
14	chr21	27140320	27141163	TSS	ADAMTS1	-721
15	chr21	32599599	32600267	Intron	MRAP	13604
16	chr21	37024917	37025922	Intron-Exon	SIM2	31056
17	chr21	45319933	45320723	TSS	C21orf122	-2379
18	chr21	42875768	42876797	TES	SLC37A1	82957
19	chr21	45445892	45447432	Intron	ADARB1	126971
20	chr21	45057662	45058493	Intron-Exon	SUMO3	3979
21	chr21	43591708	43592550	Intergenic	SIK1	78880
22	chr21	41509869	41511027	Intron	BACE2	48271
23	chr21	44376161	44377006	TSS	C21orf33	-916
24	chr21	37038942	37040111	TES	SIM2	45081
25	chr21	41906359	41907177	Intergenic	NCRNA00111	-65354
1	chr22	45864893	45866358	Intron	TBC1D22A	327680
2	chr22	14722702	14723328	Intergenic	POTEH	-54765
3	chr22	14718625	14721614	Intergenic	POTEH	-50688
4	chr22	19448709	19449440	Intron-Exon	PI4KA	-94361
5	chr22	15461100	15461862	TSS	psiTPTE22	-939
6	chr22	14716524	14717433	Intergenic	POTEH	-48587
7	chr22	36805697	36806548	Intron	SLC16A8	2568
8	chr22	15998740	15999691	Intron-Exon	CECR5	20478
9	chr22	39903812	39904786	Exon	EP300	85252
10	chr22	36154248	36156001	TSS	ELFN2	-797
11	chr22	16770596	16771487	Intron	MICAL3	115838
12	chr22	16665961	16666965	Intron	MICAL3	220360
13	chr22	47262347	47263054	TSS	FAM19A5	-898
14	chr22	47031241	47032263	Intergenic	FAM19A5	-231689
15	chr22	44001023	44002200	Intron	C22orf9	13114
16	chr22	18675688	18676880	Intergenic	DGCR6L	10728
17	chr22	40653310	40654087	TSS	TNFRSF13C	-543
18	chr22	23931058	23931790	Intron-Exon	CRYBB3	5233
19	chr22	39960215	39962431	TES	L3MBTL2	28956
20	chr22	19119082	19120231	Intron	SCARF2	1915
21	chr22	17893177	17894970	TSS	CLDN5	-317
22	chr22	19122731	19123658	TSS	SCARF2	-585
23	chr22	37653460	37654935	Intergenic	APOBEC3A	-28538
24	chr22	29657937	29658749	Intron-Exon	MORC2	35438
25	chr22	39975872	39977196	Intron-Exon	RANGAP1	34966

a, Chromosomal coordinates are derived from the UCSC hg18 genome build

b, TSS, overlaps a 3 kbp region upstream of the transcriptional start site of a gene; TES, overlaps a 3 kbp region dowstream of the transcriptional termination site of a gene; Exon, region is entirely contained within a gene exon; Intron, region is entirely contained within a gene intron; Intron-Exon, region overlaps an intron-exon boundary

c, Gene associated with the annotation shown in the Gene annotation column. In the case of regions annotated as intergenic, the nearest gene is shown.

d, Distance (in bp) to the transcriptional start site of the gene shown in the Gene name column. If region falls upstream of the TSS, then this will be a negative integer; if region falls downstream of the TSS, then this will be a positive integer; if region overlaps the TSS, then the value is zero.
