# Supplementary material for: Chromosome-wide mapping of DNA methylation patterns in normal and malignant prostate cells reveals pervasive methylation of gene-associated and conserved intergenic sequences
Source: BMC Genomics. 2011 Jun 13;12:313. doi: 10.1186/1471-2164-12-313 (PMC3124442; doi:10.1186/1471-2164-12-313)
Supplement: Additional file 10 — Primers for bisulfite genomic sequencing, COMPARE-MS, and analysis of MBD2-MBD enrichment. [file 1471-2164-12-313-S10.PDF]

Additional file 10.

Bisulfite genomic sequencing primers

MBD-Chip Region	BSF-seq Region	Strand	Forward primer	Reverse primer	Ta [1]
Chr21: 26293990-26294605	Chr21: 26294130-26294473	minus	TTTAGGTTGTTATTGGGAGGATTAA	TCTATTACAAAAAACAAAATCAATAAC	52
Chr21: 33848406-33849991	Chr21: 33848948-33849263	plus	AAGAAAGAGATTATTTAAGTTTAAGTTTTA	AAAAATACTAAAACTCCTTCTTCTACCC	52
Chr22: 15463678-15464023	Chr22: 15463837-15464163	plus	AAGAGTATTTATTTAGAGAGAGTGTAGATG	ACAATTCAATAAAAATATCATCTCATAAAA	53
Chr21: 41761264-41761600	Chr21: 41760473-41761626	plus	TATTTTGTTTGTGTATGGTATTTGT	ACCTCAAAACTTAACCCCTTAATAC	52
Chr21: 42997102-42997914	Chr21: 42997104-42997440	minus	AAATGTAGTTTTTTTGGATAGTTAG	AAAACAAAAAACAATACTACACC	52
Chr21: 42048840-42050404	Chr21: 42049667-42049988	minus	TATTTTGTTTGTGTATGGTATTTGT	ACCTCAAAACTTAACCCCTTAATAC	52
Chr21: 45109722-45111946	Chr21: 45109950-45110257	plus	TAAGTTTTTTTTAAGATATGTGGAA	AAATCCAAAAACACAAATATACTAC	52
Chr21: 45445892-45447432	Chr21: 45446849-45447160	plus	GTGGGTATTGTTAGGTAGAGGTTGT	AAAAAAATACTCAACACAATATAACAC	52
Chr21: 45098600-45099777	Chr21: 45099319-45099583	minus	TTTGAGAAAATAGATTTTATGAAGA	ACAAAACATAATAAAATCAAAAAAA	52
Chr21: 27140320-27141163	Chr21: 27140626-27140932	plus	TTTTTTTTATTAGGAAGGGAAAAGG	CCAAATCTACTTAAAACCAACCAAA	55
Chr21: 45319933-45320723	Chr21: 45320067-45320465	plus	GTGTTTTTGTTTAGGTTTTTATGT	AATCATCAAAAAACAATTAACTTTTCTATC	53
Chr21: 37273776-37274673	Chr21: 37274421-37274720	plus	GGTTATAAAATGATAAAGTTTTAAATAGAG	AATTTCCTAAAAACTTACCAAATTCC	53
Chr21: 37514464-37515930	Chr21: 37515261-37515610	plus	AGGATGGGTAGGATTTGTTT	ATATTCTCAATTCCTTCCTACTTCC	53
Chr21: 46541984-46543950	Chr21: 46542325-46542587	plus	TTTTTTGTTTTTATTTTTTGAGTATTGG	AACATTTTCATATCCCTAAATCATTTC	Step Down
Chr21: 45888804-45889875	Chr21: 45888581-45888880	plus	GAGAAGAGATTTGGGGAATATTT	AAAAAACATCACCAAACAAAAA	Step Down
Chr22: 38248790-38249522	Chr22: 38248536-38248829	plus	AAGAAAAGGGTTTTTTAGTTGAGGA	CTCCAAAACCCCATAAATCAAC	Step Down
Chr22: 15461100-15461862	Chr22: 15461752-15462138	minus	GAGTTAAGTTGTGGATTTAGAGATT	AAAATCAAATAATTAAATTTTTTATTAACTT	53
Chr22: 20442556-20443568	Chr22: 20442814-20442999	plus	TGTTTTTTTATAGATAGTTTAGATTGG	AAAAAAACCAAATAAAATACAACCAC	Step Down
Chr22: 36154248-36156001	Chr22: 36154884-36155283	plus	TTTGGGGTATGTAGAAGGATTATTG	CTACTCAATTTCTTAAACAAAAAC	50
Chr21: 41974511-41975857	Chr21: 41974722-41974935	plus	ATAGGAAGGGAAAGAAAATAGTTTG	ACCTACCATAAATCCTTAACCCTAC	Step Down
Chr22: 47262347-47263054	Chr22: 47262141-47262464	plus	GGGGTGGTGTTGTGGTAGTTAT	CTCCTAATTCATCTCCCTAAAATCC	55
Chr22: 19122731-19123658	Chr22: 19122999-19123349	plus	AGAGGGATAGGTTTTAGGGAGTTAG	TTAAACCTTTCAATAACTCCTCCTC	55

[1], Annealing temperature in degrees Celsius. Step Down indicates Step Down PCR with cycling conditions as follows: 95°C for 3 min; followed by 7 cycles of 95°C for 30 s, step down by 1°C in each cycle from 57 to 50°C for 45 s, and 72°C for 45 s; followed by 32 cycles of 95°C for 30 s, 50°C for 45 s, and 72°C for 45 s.

Primers for COMPARE-MS and analysis of MBD2-MBD enrichment

Region	Forward Primer	Reverse Primer
ADAMTS1_Up	GGAAGGACGTGTTTCACAGG	ACTTGGAGCCAACCAAGAGA
SCARF2_Up	GTGCTGTGGAGTTGGCTTTC	CCTCCGCTCCCTAGACATC
DSCR9_Down	GCGTATAGACGAGCCCATGT	CGCATTTTCCCATCCTCTT
C21orf57_Down	GAGCAGCACCTCAGAGACCT	CAGTGGGAAGGCATTTTCAT
HLCS_Intra	GCCGTGGAATCTGGTAAGTT	GGTATCAGGCCTGCAGGTAG
GSTP1	GGGACCCTCCAGAAGAGC	ACTCACTGGTGGCGAAGACT
PTGS2	GGAGAGGAAGCCAAGTGTCC	GGTTTCCGCCAGATGTCTTT
HBB	GCAACCTCAAACAGACACCA	CCTCACCACCAACTTCATCC
